# Examining the replicability of online experiments selected by a decision market

**DOI:** 10.1038/s41562-024-02062-9

**Published:** 2024-11-19

**Authors:** Felix Holzmeister, Magnus Johannesson, Colin F. Camerer, Yiling Chen, Teck-Hua Ho, Suzanne Hoogeveen, Juergen Huber, Noriko Imai, Taisuke Imai, Lawrence Jin, Michael Kirchler, Alexander Ly, Benjamin Mandl, Dylan Manfredi, Gideon Nave, Brian A. Nosek, Thomas Pfeiffer, Alexandra Sarafoglou, Rene Schwaiger, Eric-Jan Wagenmakers, Viking Waldén, Anna Dreber

**Affiliations:** 1 Department of Economics, University of Innsbruck, Innsbruck, Austria; 2 Department of Economics, Stockholm School of Economics, Stockholm, Sweden; 3 Division of the Humanities and Social Sciences, California Institute of Technology, Pasadena, CA USA; 4 John A. Paulson School of Engineering and Applied Sciences, Harvard University, Boston, MA USA; 5 Nanyang Technological University, Singapore, Singapore; 6 Faculty of Social and Behavioural Sciences, Utrecht University, Utrecht, The Netherlands; 7 Department of Banking and Finance, University of Innsbruck, Innsbruck, Austria; 8 Institute of Social and Economic Research, Osaka University, Osaka, Japan; 9 Lee Kuan Yew School of Public Policy, National University of Singapore, Singapore, Singapore; 10 Faculty of Social and Behavioural Sciences, University of Amsterdam, Amsterdam, The Netherlands; 11 Machine Learning, Centrum Wiskunde and Informatica, Amsterdam, The Netherlands; 12 Independent Researcher, Vienna, Austria; 13 Marketing Department, Wharton School, University of Pennsylvania, Philadelphia, PA USA; 14 Department of Psychology, University of Virginia, Charlottesville, VA USA; 15 Center for Open Science, Charlottesville, VA USA; 16 Institute for Advanced Study, Massey University, Auckland, New Zealand; 17 Sveriges Riksbank, Stockholm, Sweden

**Keywords:** Economics, Human behaviour

## Abstract

Here we test the feasibility of using decision markets to select studies for replication and provide evidence about the replicability of online experiments. Social scientists (*n* = 162) traded on the outcome of close replications of 41 systematically selected MTurk social science experiments published in PNAS 2015–2018, knowing that the 12 studies with the lowest and the 12 with the highest final market prices would be selected for replication, along with 2 randomly selected studies. The replication rate, based on the statistical significance indicator, was 83% for the top-12 and 33% for the bottom-12 group. Overall, 54% of the studies were successfully replicated, with replication effect size estimates averaging 45% of the original effect size estimates. The replication rate varied between 54% and 62% for alternative replication indicators. The observed replicability of MTurk experiments is comparable to that of previous systematic replication projects involving laboratory experiments.

## Abstract (web summary)

This study finds that decision markets can be a useful tool for selecting studies for replication. For a sample of 26 online experiments published in PNAS selected by a decision market, the authors find replication rates ranging between 54% and 62%.

## Main

Can published research findings be trusted? Unfortunately, the answer to this question is not straightforward, and the credibility of scientific findings and methods has been questioned repeatedly^1–9^. A vital tool for evaluating and enhancing the reliability of published findings is to carry out replications, which can be used to sort out likely true positive findings from likely false positives. A replication essentially updates the probability of the hypothesis being true after observing the replication outcome. A successful replication will move this probability towards 100%, while a failed replication will move it towards 0% (refs. ^10,11^). In recent years, several systematic large-scale replication projects in the social sciences have been published^12–17^, reporting replication rates of around 50% in terms of both the fraction of statistically significant replications and the relative effect sizes of replications. Potential factors to explain these replication rates may be low statistical power^1,18,19^ in the original studies, testing original hypotheses with low priors^1,10,20^ and questionable research practices^1,21,22^. Systematic replication studies led to discussions about improving research practices^23,24^ and have substantially increased the interest in independent replications^25^. However, as it is time-consuming and costly to conduct replications, it has been argued that it is useful to have a principled mechanism to decide which replications to prioritize to facilitate efficient and effective usage of resources^25–37^. Here we test the feasibility of one potential method to select which studies to replicate. Building on previous work using prediction markets^38–40^ to forecast replicability, we adapt the forecasting methodology to what is referred to as decision markets^41–44^.

The decisive distinction between prediction markets and decision markets is that prediction markets elicit aggregate-level replicability forecasts on a predetermined set of studies, whereas decision market forecasts determine which studies are going to be put to a replication test. While previous studies provide evidence that prediction market forecasts are predictive of replication outcomes^10,16,17,45^, prediction efficiency might not generalize to decision markets, which involve more complex procedures and incentives. The performance of decision markets as a tool for selecting which empirical claims to replicate has not been systematically examined. Note that a decision market in itself is not sufficient to provide a mechanism to select studies for replication, but it has to be combined with an objective function of which studies to replicate (an example of an objective function would be to replicate the studies with the lowest probability of replication). For decision markets to be potentially useful for selecting studies for replication, it first has to be established that the predictions of the decision markets are associated with the replication outcomes. To provide such a ‘proof of concept’ of using a decision market as a mechanism to determine which studies to replicate, we first identified all social science experiments published in the Proceedings of the National Academy of Sciences (PNAS) between 2015 and 2018 that fulfilled our inclusion criteria for (1) the journal and period; (2) the platform on which the experiment was performed (Amazon Mechanical Turk; MTurk); (3) the type of design (between-subjects or within-subject treatment design); (4) the equipment and materials needed to implement the experiment (the experiment had to be logistically feasible for us to implement); and (5) the results reported in the experiment (at least one main or interaction effect with *P* < 0.05 reported in the main text). On the basis of our inclusion criteria, we identified 44 articles, 3 of which have been excluded owing to a lack of feasibility, leaving us with a final sample of 41 articles^46–86^ (see Methods for details on the inclusion criteria). For each of these articles, we identified one critical finding with *P* < 0.05 that we could potentially replicate (see Methods for details and Supplementary Table 1 for the hypotheses selected for each of the 41 studies).

We then invited social science researchers to participate as forecasters in both a prediction survey and an incentivized decision market on the 41 studies. In the survey, the forecasters independently estimated the probability of replication for the 41 studies. In the decision market, they could trade on whether the result of each of the 41 studies would replicate. Participants in the decision market received an endowment of 100 tokens corresponding to USD 50, and 162 participants made a total of 4,412 trades. Traders in the market were informed about the preregistered decision mechanism: the 12 studies with the highest and the 12 studies with the lowest market prices were to be selected for close replication; in addition, 2 randomly chosen studies (out of the remaining 17 studies) are replicated to ensure incentive compatibility, with participant payoffs scaled up by the inverse of their probability in the decision rule (see Methods for details). For incentive compatibility, all the 41 studies included need to have a strictly positive probability of being selected for replication, which is ensured by having at least one randomly selected study. Otherwise, traders would be incentivized to only trade on those studies that will most likely be chosen according to the decision rule.

All replication experiments, just like all original studies, were conducted on Amazon Mechanical Turk (MTurk), and the same sample restrictions and exclusion criteria as the original studies were applied, which guards against concerns about the potential moderating effects of culture differences in replications^14,87^. Replication sample sizes were determined to have 90% power to detect 2/3 of the effect size reported in the original study at the 5% significance level in a two-sided test (with the effect size estimates having been converted to Cohen’s *d* to have a common standardized effect size measure across the original studies and the replication studies; see Methods for details). If sample size calculations led to replication sample sizes smaller than in the original study, we targeted the same sample size as in the original study. The average sample size in the replications (*n* = 1,018) was 3.5 times as large as the average sample size in the original studies (*n* = 292).

The replication results for the 26 MTurk experiments selected by the decision market constitute the second contribution of this project. Systematic evidence on the replicability of online experiments in the social sciences is lacking, and concerns about the quality of online experiments in general—and MTurk studies in particular—have been raised^88–94^. Needless to say, the replication results only pertain to the single focal result selected per paper, and the replication outcome does not necessarily generalize to other results reported in the original articles^95,96^. For convenience, we refer to the replications as ‘replication of [study reference]’ though. Also, our assessment of the most central result may differ from that of the original authors.

Preregistering study protocols and analysis plans have been proposed as a means to reduce questionable research practices. While empirical evidence is still limited, some recent studies suggest that these practices enhance the credibility of published findings^97–99^, although potential issues with preregistration have also been raised^100–102^. Before starting the survey data collection (that preceded the decision market and replications), we preregistered^103,104^ an analysis plan (‘replication report’) for each of the 41 potential replications at OSF after obtaining feedback from the original authors (https://osf.io/sejyp). After the replications had been conducted, the replication reports of the 26 studies selected for replication were updated with the results of the replications (and potential deviations from the protocol) and were posted to the same OSF repository. We also preregistered an overall analysis plan at OSF before starting the data collection, detailing the study’s design and all planned analyses and tests (https://osf.io/xsp6g). Unless explicitly stated, all analyses and tests reported in the paper have been preregistered and adhere exactly to our preregistered analysis plan. Supplementary Notes details any deviations from the planned design and analyses for the 26 replications.

We preregistered two primary replication indicators and two primary hypotheses. The two primary replication indicators are the relative effect size of the replications and the statistical significance indicator for replication (that is, whether or not the replication results in a statistically significant effect with *P* < 0.05 in the same direction as the original effect), which was the replication outcome predicted by forecasters in the survey and the decision market.

The statistical significance indicator is a binary criterion of replication and is based on testing the hypothesis for which the original study found support using standard null hypothesis significance testing. The indicator crudely classifies replications as failed or successful depending on whether the replication study yields evidence in support of the original hypothesis at a particular significance threshold. (Critics of null hypothesis significance testing or privileging a *P* value of 0.05 will, justifiably so, object to this crude classification; that is why it is only one of the several indicators that we report.) A replication classified as failed based on this indicator, however, does not imply that the estimated replication effect size is significantly different from the original estimate (see more on this below). To keep the false negative risk at bay and to be informative, the statistical significance indicator calls for well-powered replications (as in this study)^105,106^. However, a limitation of this indicator for well-powered replication studies is that it may classify a replication as successful even if the observed effect size is substantially smaller (or larger) than in the original study. While the statistical significance indicator dichotomizes replication outcomes into successful and failed, replicability may be perceived as a continuous matter of degree. This is why we also consider the relative effect size—a continuous measure of replicability—as a primary replication indicator. While the relative effect size constitutes an imprecise indicator for an individual replication study, it arguably provides an informative measurement of the extent of replicability for a group of studies as it quantifies the average degree of apparent inflation in the original effect sizes^107^. As all replication indicators have limitations, we preregistered four additional secondary replication indicators. In addition, we report the results for two non-preregistered replication indicators, which were helpfully suggested during the review process of the paper.

In our two primary hypotheses, we conjecture that (1) the decision market prices positively correlate with the replication outcomes and (2) the standardized effect sizes in the replications are lower than in the original studies. All hypotheses are evaluated using two-tailed tests, and—following Benjamin et al.^108^—we interpret results with *P* < 0.005 as ‘statistically significant evidence’, whereas results with 0.005 ≤ *P* < 0.05 are considered ‘suggestive evidence’. No adjustments were made for multiple comparisons.

## Results

### Replication outcomes and decision market performance

**Fig. 1:**
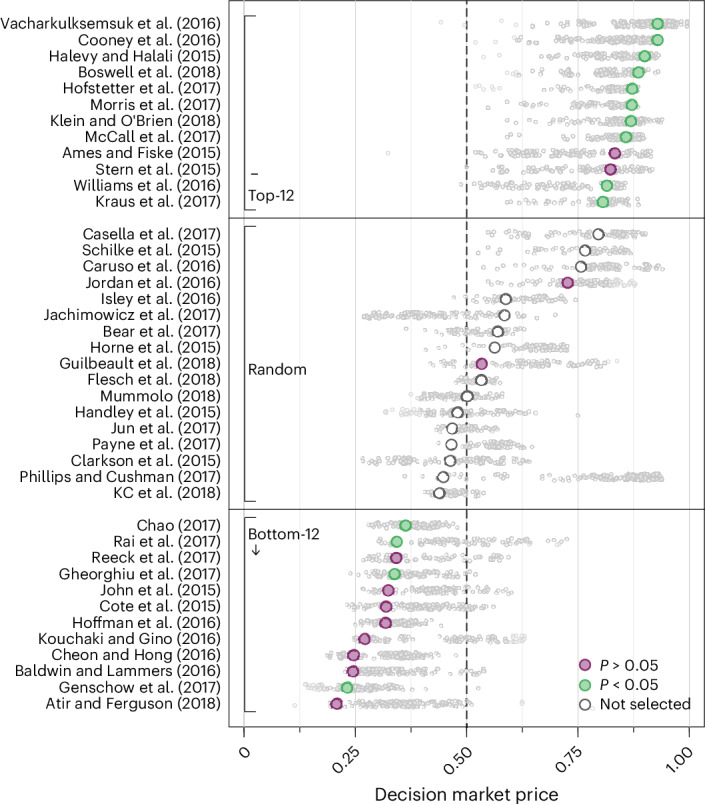
Decision market prices for the 41 included studies. Plotted are the decision market prices for the 41 MTurk social science experiments published in PNAS between 2015 and 2018. The small grey dots indicate the market prices after each market transaction; the larger dots indicate the final market price. The studies are ordered based on the final decision market prices, which can be interpreted as the market’s probability forecast of successful replication. The 12 studies with the highest decision market prices and the 12 studies with the lowest decision market prices were selected for replication; in addition, 2 of the remaining 17 studies were selected for replication at random to ensure that the decision market is incentive compatible. The replication outcomes for the statistical significance indicator are also illustrated for the 26 replicated studies. The point-biserial correlation between the decision market prices and the replication outcomes in primary hypothesis 1 is *r* = 0.505 (95% CI (0.146, 0.712), *t*(24) = 2.867, *P* = 0.008; *n* = 26, two-sided test).

**Fig. 2:**
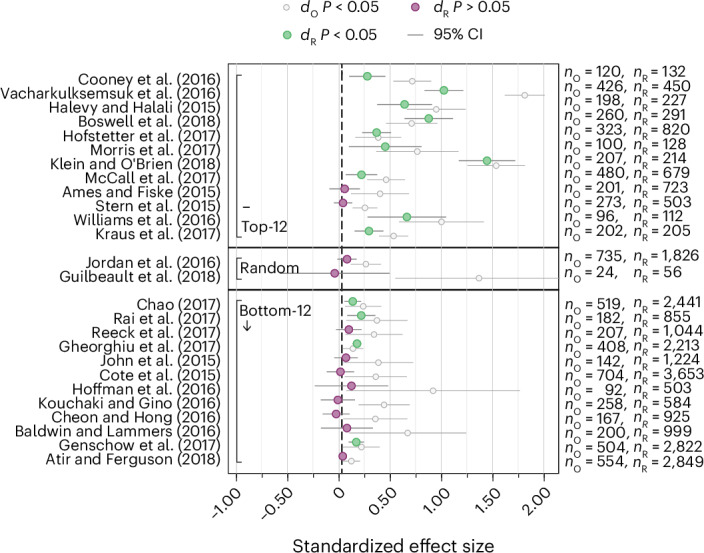
Replication results. Plotted are the point estimates and the 95% CIs (standardized to Cohen’s *d* units) of the 26 replications (*d*_R_) and original studies. Studies within each of the three panels (top-12, random, bottom-12) are sorted based on the decision market prices as in Fig. 1. There is a statistically significant effect (*P* < 0.05) in the same direction as the original study for 14 out of 26 replications (53.8%; 95% CI (33.4%, 73.4%)). For the 12 studies with the highest decision market prices, there is a statistically significant effect (*P* < 0.05) in the same direction as the original study for 10 out of 12 replications (83.3%; 95% CI (51.6%, 97.9%)). For the 12 studies with the lowest decision market prices, there is a statistically significant effect (*P* < 0.05) in the same direction as the original study for 4 out of 12 replications (33.3%; 95% CI (9.9%, 65.1%)). Our secondary hypothesis test provides suggestive evidence that the difference in replication rates between the top-12 and the bottom-12 group is different from zero (Fisher’s exact test; *χ*^2^(1) = 6.171, *P* = 0.036; *n* = 24, two-sided test). The error bars denote the 95% CIs of the original and the replication effect size estimates. The numbers of observations used to estimate the 95% CIs are the original and replication sample sizes noted on the right as *n*_O_ and *n*_R_.

Figure 1 and Supplementary Table 2 show the results for the decision markets where the final market price can be interpreted as the predicted replication probability. The predicted probabilities of replication range from 20.9% to 92.9% for the 41 studies, with a mean of 57.6% (s.d. = 23.6%). The average predicted probability for the 26 studies eventually selected for replication is 58.5%. Figure 1 also delineates the replication outcomes based on the statistical significance indicator, which allows for gauging the relationship between the decision market prices and the replication outcomes. In Fig. 2 and Supplementary Table 3, we show the replication results for the 26 studies selected for replication. Of the 26 claims, 14 (53.8%; 95% confidence interval (CI) (33.4%, 73.4%)) replicated successfully according to the statistical significance indicator. The point-biserial correlation between decision market prices and the binary replication outcome, testing our first primary hypothesis, is *r* = 0.505 (95% CI (0.146, 0.712); *t*(24) = 2.867, *P* = 0.008; *n* = 26). Thus, in support of our first primary hypothesis, we find suggestive evidence of a positive association between decision market prices and replication outcomes. As a related secondary hypothesis, we test if the replication rate is lower among the 12 studies with the lowest decision market prices than for the 12 studies with the highest decision market prices. The replication rate is 33.3% (95% CI (9.9%, 65.1%)) for the studies in the ‘bottom-12’ group and 83.3% (95% CI (51.6%, 97.9%)) for the studies in the ‘top-12’ group, yielding suggestive evidence in support of our secondary hypothesis (Fisher’s exact test; *χ*^2^(1) = 6.171, *P* = 0.036; *n* = 24). Note Fisher’s test conditions on the margin totals; hence, it is only exact for the conditional distribution and can be overly conservative if the margin totals are unknown^109,110^, as is the case in our analysis. Boschloo’s test^111^, an exact unconditional procedure uniformly more powerful than Fisher’s test, also yields suggestive evidence for the difference in proportions between the ‘top-12’ and the ‘bottom-12’ group (not preregistered; 95% CI (0.089, 0.799), *P* = 0.017; *n* = 24).

### Relative effect sizes

**Fig. 3:**
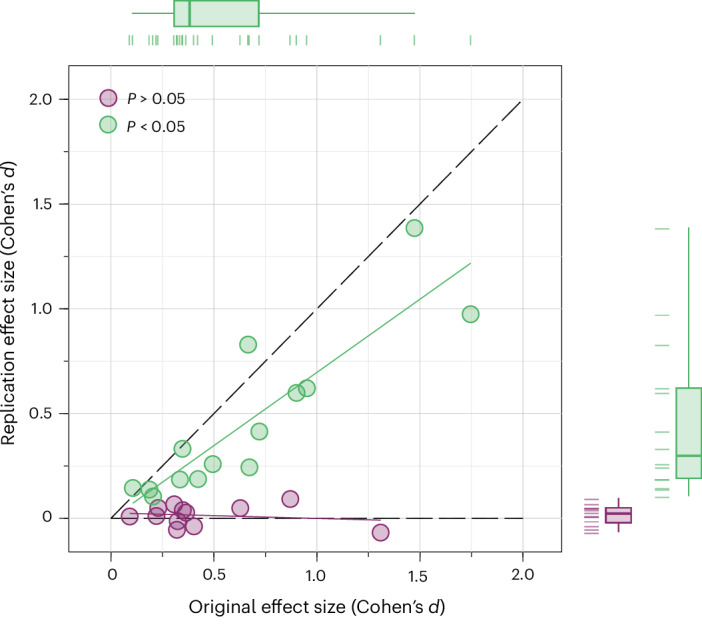
Relationship between estimated original and replication effect sizes. Plotted are the estimated original and replication effect sizes for each of the 26 replication studies (the estimated effect sizes of both the original and replication studies are standardized to Cohen’s *d* units). The 95% CIs for the original and replication effect size estimates are illustrated in Fig. 2 and tabulated in Supplementary Table 3. The mean estimated effect size of the 26 replication studies is 0.253 (s.d. = 0.357) compared with 0.563 (s.d. = 0.426) for the original studies, resulting in a relative estimated average effect size of 45.0%, confirming our second primary hypothesis (Wilcoxon signed-rank test, *z* = 4.203, *P* < 0.001; *n* = 26, two-sided test). The estimated relative effect size of the 13 replications that have been successfully replicated according to the statistical significance indicator is 69.5%, and the estimated relative effect size of the 13 studies that did not replicate is 3.2%. The box plots show the median, the interquartile range, and the 5th and 95th percentile of the effect size estimates in the 26 original studies and the 26 replication studies.

The mean estimated effect size of the 26 replication studies (in Cohen’s *d* units) is 0.253 (s.d. = 0.357) compared with 0.563 (s.d. = 0.426) for the original studies, implying a relative estimated average effect size, just dividing the two numbers, of 45.0%; the difference in estimated effect sizes is statistically significant, supporting our second primary hypothesis of systematically smaller estimated effect sizes in the replications (Wilcoxon signed-rank test, *z* = 4.203, *P* < 0.001; *n* = 26). The relative effect size can also be estimated for each study separately (reported in Supplementary Table 3) and varies between −17.0% and 136.2%, with a mean estimate across studies of 41.1% (95% CI (24.5%, 57.7%)). For the 14 studies that replicated according to the statistical significance indicator, the first and the second relative effect size measures as defined above are 69.5% and 72.0% (95% CI (54.8%, 89.3%)), indicative of some inflation in original effect sizes even for apparent true positives. The two estimated relative effect size measures for the 12 studies that failed to replicate according to the statistical significance indicator are 3.2% and 5.0% (95% CI (−2.6%, 12.5%)), respectively. Figure 3 illustrates the relationship between the estimated original and replication effect sizes.

### Secondary replication indicators

**Fig. 4:**
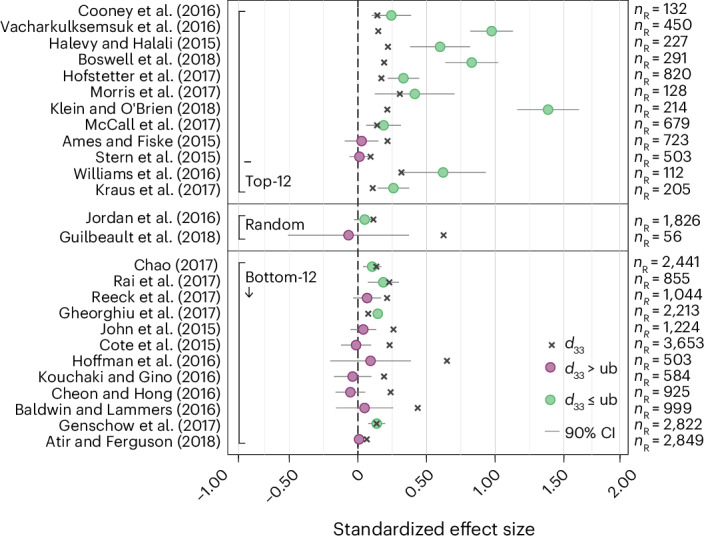
Replication results based on the small-telescopes approach (a secondary replication indicator). Plotted are the 90% CIs of replication effect sizes in relation to small-effect sizes as defined by the small-telescopes approach^112^ (the effect size that the original study would have had 33% power to detect). Studies within the three panels (top-12, random, bottom-12) are sorted based on the decision market prices as in Fig. 1. A study is defined as failing to replicate if the 90% CI is below the small effect (with ‘ub’ denoting the upper bound of the 90% CI). According to the small-telescopes approach, 15 out of 26 studies (57.7%; 95% CI (36.9%, 76.6%)) replicate. The error bars denote the 90% CIs of the estimated replication effect sizes. The numbers of observations used to estimate the 90% CIs are the replication sample sizes noted on the right as *n*_R_.

**Fig. 5:**
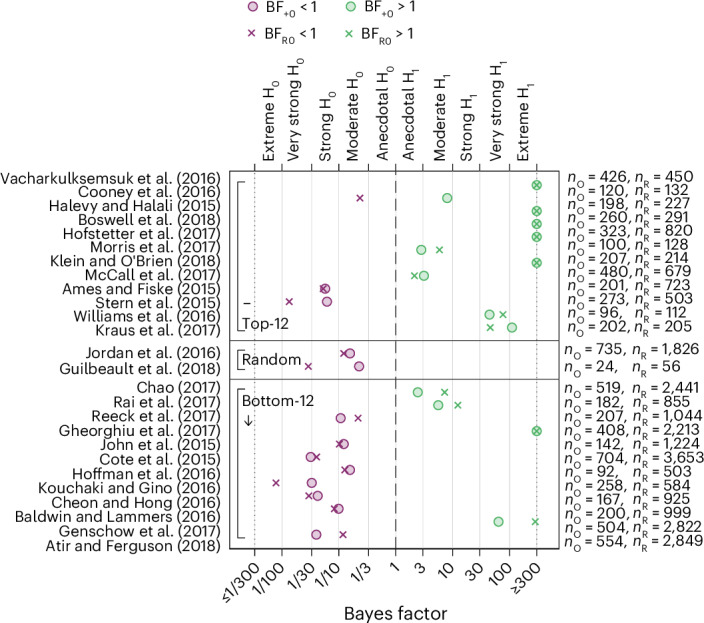
Replication results based on Bayes factors (secondary replication indicators). The figure plots the one-sided default Bayes factor (BF_+0_) and the replication Bayes factor (BF_R0_) for the 26 replications^113^. BF_+0_ > 1 favours the hypothesis of an effect in the direction of the original paper, whereas BF_+0_ < 1 favours the null hypothesis of no effect. BF_R0_ quantifies the additional evidence provided by the replication results on top of the original evidence. BF_R0_ > 1 indicates additional evidence in favour of the alternative over the null, whereas BF_R0_ < 1 indicates additional evidence for the null instead. The evidence categories proposed by Jeffreys^115^ are also shown (from extreme support for the null hypothesis to extreme support for the original hypothesis). Studies within the three panels (top-12, random, bottom-12) are sorted based on the decision market prices as in Fig. 1. The BF_+0_ is above 1 for all 14 replication studies that successfully replicated according to the statistical significance indicator and below 1 for all 12 replication studies that failed to replicate according to the statistical significance indicator. The BF_R0_ is above 1 for 13 of the 14 replication studies that replicated according to the statistical significance indicator and below 1 for Cooney et al.^56^ whose estimated relative effect size of 0.36 is the lowest among these 14 studies; the BF_R0_ is below 1 for all of the 12 replication studies that failed to replicate according to the statistical significance indicator. The numbers of observations used to estimate BF_+0_ and BF_R0_ are the original and replication sample sizes noted on the right as *n*_O_ and *n*_R_.

**Fig. 6:**
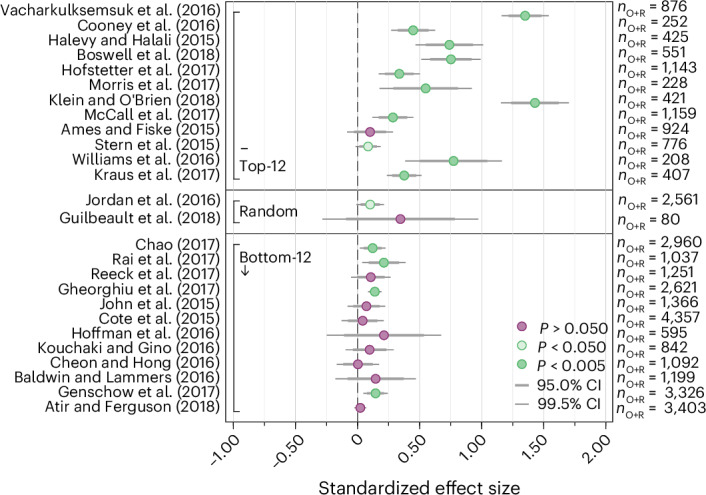
Meta-analytic estimated effect sizes combining the original and the replication estimated effect sizes (a secondary replication indicator). The figure plots the point estimates and 95% and 99.5% CIs of the fixed-effects weighted meta-analytic effect sizes, combining the original and the replication studies (standardized to Cohen’s *d* units). Studies within the three panels (top-12, random, bottom-12) are sorted based on the decision market prices as in Fig. 1. As preregistered, we report the significance of the estimated meta-analytic effect sizes for both the 0.05 significance threshold and the 0.005 significance threshold (based on a two-sided *z*-test). Sixteen out of 26 (61.5%; 95% CI (40.6%, 79.8%)) studies replicated according to the statistical significance indicator using the 0.05 significance threshold, and 14 out of 26 (53.8%; 95% CI (33.4%, 73.4%)) studies replicated using the 0.005 significance threshold. The error bars denote the 95% CIs of the estimated meta-analytic effect sizes. The number of observations used to estimate the 95% CIs are the sums of the original and replication sample sizes noted next to the study identifier on the *y*-axis as *n*_O+R_.

We also preregistered four secondary replication indicators: the small-telescopes approach^112^, the one-sided default Bayes factor^113^, the replication Bayes factor^114^ and the fixed-effects weighted meta-analytic effect size (see Methods for details). When relying on the small-telescopes approach, testing if the replication effect size is smaller than a ‘small effect’^112^, 15 studies (57.7%; 95% CI (36.9%, 76.6%)) are considered successful replications (Fig. 4 and Supplementary Table 4). The one-sided default Bayes factor (BF_+0_) indicates the strength of evidence in favour of the alternative hypothesis as opposed to the null hypothesis. BF_+0_ exceeds 1 for the 14 studies (53.8%; 95% CI (33.4%, 73.4%)) that replicated according to the statistical significance indicator, with strong evidence (BF_+0_ > 10) for the tested hypothesis for 9 studies (34.6%; 95% CI (17.2%, 55.7%)); BF_+0_ is below 1 for the 12 replications (46.2%; 95% CI (26.6%, 66.6%)) that failed to replicate according to the statistical significance indicator, with strong evidence (BF_+0_ < 0.1) for the null hypothesis for 7 studies (26.9%; 95% CI (11.6%, 47.8%)) based on the evidence categories proposed by Jeffreys^115^ (Fig. 5 and Supplementary Table 4). The one-sided replication Bayes factor (BF_R0_) indicates the strength of additional evidence in favour of the alternative hypothesis as opposed to the null hypothesis, given the already acquired evidence based on the original data^114^. Replication Bayes factors lead to similar conclusions as the one-sided default Bayes factors, with BF_R0_ > 10 for 10 studies (38.5%; 95% CI (20.2%, 59.4%)) and BF_R0_ < 0.1 for 7 studies (26.9%; 95% CI (11.6%, 47.8%)). One exception to this is the study by Cooney et al.^56^, for which the default Bayes factor exceeds one (BF_+0_ = 8.01) but the replication Bayes factor is below one (BF_R0_ = 0.23) owing to the replication effect size being only about a third of the original effect size and a larger sample size in the replication compared with the original study (Fig. 5 and Supplementary Table 4). The meta-analytic effect size is statistically significant at the 5% level for 16 studies (61.5%; 95% CI (40.6%, 79.8%)) and significant at the 0.5% level for 14 studies (53.8%; 95% CI (33.4%, 73.4%)) (Fig. 6 and Supplementary Table 4). The meta-analytic effect sizes should be interpreted cautiously as original effect sizes reported as statistically significant are likely to be overestimated on average owing to insufficient sample sizes and, thereby, statistical power (and potentially owing to questionable research practices)^18,19^. Overall, the primary and secondary replication indicators yield the same binary conclusions for 23 of the 26 replications.

### Non-preregistered replication indicators

**Fig. 7:**
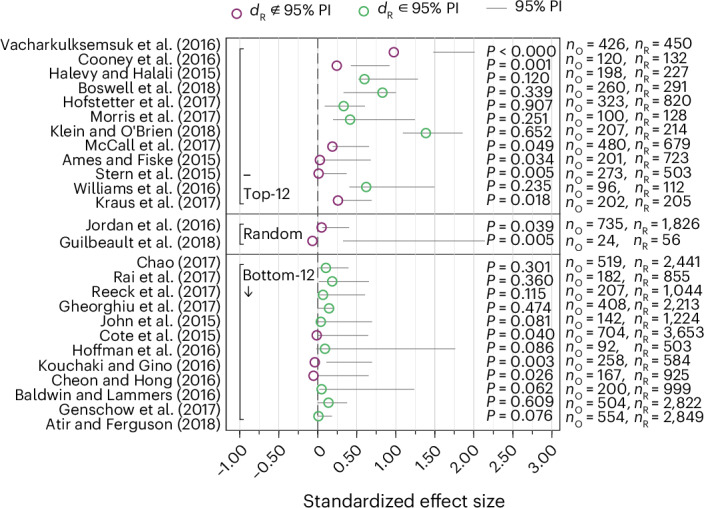
Replication results based on prediction intervals (not preregistered). Plotted are the 95% prediction intervals^116^ (PIs) for the standardized original effect sizes (Cohen’s *d*). Studies within the three panels (top-12, random, bottom-12) are sorted based on the decision market prices as in Fig. 1. Fifteen replications out of 26 (57.7%; CI (36.9%, 76.6%)) are within the 95% prediction interval and replicate according to this indicator. The *P* values reported on the right are based on two-sample *z*-tests for a difference between the replication effect size and the original effect size. The grey lines denote the 95% prediction intervals, and the small circles denote the mean replication effect sizes. All tests are two-sided. The numbers of observations used to estimate the 95% prediction intervals are the original and replication sample sizes noted next to the study identifier on the *y*-axis as *n*_O_ and *n*_R_.

Following the suggestion of a reviewer, we report the results for two additional replication indicators. The first alternative replication indicator is a test of whether the replication effect size is statistically significantly different from the original effect size. This indicator is closely related to the prediction interval approach^116^ as the results can be illustrated as prediction intervals that the replication effect sizes are evaluated against: if the replication effect size falls outside the 95% prediction interval, the replication and original effect sizes differ at the 5% significance level, and the replication is considered a failure. The prediction interval approach, thus, yields a binary replication indicator, which is complemented by a continuous replicability measure defined as the *P* value of the test of a significant difference between the replication and original effect sizes. We illustrate the prediction interval results in Fig. 7 and report the *z*-statistics and *P* values in Supplementary Table 5 (the *P* values are also shown in Fig. 7). According to the prediction interval indicator, 15 studies (57.7%, 95% CI (36.9%, 76.6%)) replicate. This replication rate is close to the result for the statistical significance indicator. However, the classification of nine replication outcomes shifts: for four studies, the classification changes from successful to failed, and for five studies, the classification changes from failed to successful. These changes are due to the fact that low-powered original studies are more likely to replicate, whereas high-powered original studies are less likely to replicate based on the prediction interval indicator (compared with evaluating replicability based on the statistical significance indicator). According to the prediction interval indicator, six studies failed to replicate among the top-12 studies in terms of decision market prices, whereas three studies failed to replicate among the bottom-12 studies.

### Associations between indicators (not preregistered)

To examine the relationship between the replication indicators, we estimated Kendall’s rank correlations *τ*_b_ between all the replication indicators used in the study (Supplementary Table 6). All preregistered replication indicators are strongly correlated with each other, with *τ*_b_ varying between 0.61 and 1.00 (*P* < 0.005 for all correlations). However, they are more weakly to moderately correlated with the prediction interval approach and *P* values from *z*-tests comparing the replication and original effect sizes (with *τ*_b_ varying between 0.12 and 0.56).

Each of the various replication indicators presented in this study has its strengths and weaknesses. There is no general consensus about which indicator is most appropriate^15–17,117–119^. Therefore, we chose to report the results for a host of indicators and leave it to readers to judge the suitability of the different indicators and their degree of consensus. The overall replication rate is similar for all the binary replication indicators and varies between 14 (53.8%) and 16 (61.5%) studies. The agreement about which results are classified as successfully replicated is large between the indicators, with the exception of the prediction interval approach. The estimated average relative effect size of around 45%, which can be interpreted in terms of a replicability rate, yields an estimate in the same ballpark. The somewhat lower estimate for the relative effect size is due to the fact that not only the false positive rate but also the inflation of true positive effect sizes is factored in. Another advantage of the relative effect size indicator is that it is not affected by replication power. The three other continuous replication indicators cannot be aggregated across studies and are thus difficult to compare to the other indicators on an aggregated level.

### Replicability forecasts and indicators (not preregistered)

In Supplementary Table 7, we also provide Pearson correlations between our replicability forecasts (final decision market prices and average prediction survey beliefs) and all the replication indicators. Both the decision market prices and the survey beliefs are positively correlated with all the replication indicators except the prediction interval approach (although positive, the correlations to the *P* value of the test of a significant difference between the replication and original effect sizes are also close to 0). Note that these correlations should be interpreted cautiously as the forecasters predicted the replication outcomes for the statistical significance indicator but not the other replication indicators.

### Survey forecasts versus decision market predictions

**Fig. 8:**
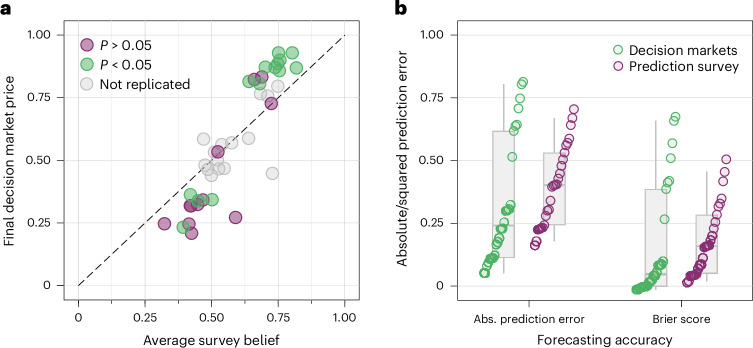
Relationship between decision market prices and mean survey beliefs and forecasting accuracy. **a**, Plotted are the decision market prices and the mean survey beliefs about replication for the 41 studies included in the decision market and the survey; the colour coding highlights the replication outcomes for the 26 replicated studies. The decision market prices and the mean survey beliefs about replication are highly correlated with a Pearson correlation of *r* = 0.899 (95% CI (0.814, 0.944); *t*(39) = 12.830, *P* = 1.4 × 10^−15^; *n* = 41, two-sided test). **b**, Plotted are the absolute prediction errors and the Brier scores (the squared prediction errors) for the decision market and the prediction survey for the 26 replicated studies. There is suggestive evidence of higher prediction accuracy for the decision market in terms of the absolute prediction error (0.353 for the decision markets and 0.421 for the survey; Wilcoxon signed-rank test, *z* = 2.172, *P* = 0.030; *n* = 26, two-sided test), but not in terms of the Brier score (0.188 for the decision markets and 0.202 for the survey; Wilcoxon signed-rank test, *z* = 1.181, *P* = 0.238; *n* = 26, two-sided test). The box plots show the median, the interquartile range, and the 5th and 95th percentile of the absolute prediction errors and Brier scores for the survey and decision market predictions of the 26 replication studies.

We tested three additional preregistered secondary hypotheses based on the survey beliefs about replication (see Methods for details and Supplementary Table 7 for the survey results). The point-biserial correlation between average survey beliefs and the replication outcomes based on the statistical significance criterion is *r* = 0.476 (95% CI (0.107, 0.694); *t*(24) = 2.650, *P* = 0.014; *n* = 26). The survey beliefs and the decision market prices are positively correlated with a Pearson correlation of 0.899 (95% CI (0.814, 0.944); *t*(39) = 12.830, *P* < 0.001; *n* = 41) (Fig. 8a). The final secondary hypothesis tests if the prediction accuracy, measured in terms of the absolute prediction error and the Brier score (that is, the squared prediction error), is higher for the decision market than the survey forecasts (Fig. 8b). The mean absolute prediction error and the mean Brier score are 0.353 and 0.188 for the decision market, and 0.421 and 0.202 for the survey, respectively, providing suggestive evidence for higher accuracy for the market forecasts based on the absolute prediction error (Wilcoxon signed-rank test, *z* = 2.172, *P* = 0.030; *n* = 26) but not the Brier score (Wilcoxon signed-rank test, *z* = 1.181, *P* = 0.238; *n* = 26). The failure to reject the null hypothesis for the Brier score does not imply that the null hypothesis is true. In the survey, we also elicited forecasters’ self-rated expertise for each study. The average self-rated expertise (of participants eventually active in the markets, *n* = 162) for the 26 replicated studies was 2.31 (s.d. = 1.40; *n* = 4,212) on a scale from 1 (‘no knowledge of the topic’) to 7 (‘very high knowledge of the topic’). Supplementary Fig. 1 plots the absolute prediction error and the Brier score of the 26 survey and decision market forecasts over the average self-rated expertise per study. We do not find evidence for the prediction accuracy and the average self-rated expertise being significantly correlated (not preregistered; see Supplementary Fig. 1 for details).

### Beliefs about the Covid-19 pandemic and replicability

A potential issue raised by some original authors in giving feedback on the replication reports before the data collection was that the replicability of some original results might be affected by the Covid-19 pandemic (as all the original studies were conducted before the pandemic). We evaluate this possibility in a preregistered exploratory analysis, relying on the forecasters’ beliefs about the impact of the pandemic on replicability. As part of the prediction survey, participants were asked to judge whether the pandemic would have affected the likelihood of successful replication, measured on a scale from −3 (‘the pandemic has definitely decreased the probability of replication’) to 3 (‘the pandemic has definitely increased the probability of replication’). We test if the average response to this question differs from zero using a one-sample *t*-test for each of the 26 replications, and we test if the average response across all 26 studies differs from 0. We find a statistically significant result for four and a suggestive result for two replications on beliefs that Covid-19 has affected the replication probability (Supplementary Table 8). For the six studies with suggestive or statistically significant evidence, the estimate is negative for two studies and positive for four; only in two of the cases does the sign of the expectation match the eventual replication outcome. For the average belief about the impact of the pandemic on replicability across the 26 studies of 162 forecasters (who were active in the decision markets), there is suggestive evidence that the mean of 0.039 (s.d. = 0.190) differs from zero (*t*(161) = 2.598, *P* = 0.010; *n* = 162). Somewhat surprisingly—and in contrast to the concerns raised by some of the original authors—there is thus a tendency for forecasters to believe that the pandemic has increased the average likelihood that the studies will replicate. However, the magnitude of the effect is small (*d* = 0.204; 95% CI (0.049, 0.360)).

In addition, we tested, estimating the point-biserial correlation, if the average belief (per study) about the pandemic’s impact on replicability correlates with the replication outcomes based on the statistical significance indicator; we do not find a statistically significant association (*r* = 0.014, 95% CI (−0.360, 0.382); *t*(24) = 0.068, *P* = 0.946; *n* = 26). Yet, we cannot rule out that Covid-19 has entailed effects on replicability not foreseen by scholars participating in the survey. Further work is needed to gauge whether and to which extent experimental replications—and predictions of replication success—might be sensitive to macro-historical secular change such as economic upheaval, wars, pandemics and so on. Forecasters’ beliefs about the pandemic’s impact on replicability are also neither statistically significantly correlated with the final decision market prices (*r* = 0.387, 95% CI (−0.008, 0.669); *t*(24) = 2.055, *P* = 0.051; *n* = 26) nor the average survey belief of replication (*r* = 0.347, 95% CI (−0.053, 0.644); *t*(24) = 1.815, *P* = 0.082; *n* = 26), although the point estimates of the correlations are quite sizeable.

### Original *P* value and replication (not preregistered)

For comparison to previous systematic large-scale replication projects, we also report the correlation between the original *P* value and the replication outcome for the statistical significance indicator. The point-biserial correlation between the original *P* value and the replication outcome for the statistical significance indicator is −0.400 (*P* = 0.043; 95% CI (0.014, 0.648)) and comparable in magnitude to correlations of −0.33 in the Replication Project: Psychology (RPP)^15^, −0.57 in the Experimental Economics Replication Project (EERP)^16^ and −0.40 in the Social Sciences Replication Project (SSRP)^17^.

## Discussion

We found suggestive evidence (*P* < 0.05) for our first primary hypothesis that final decision market prices correlate with replication outcomes (*r* = 0.505). However, the estimated effect size is somewhat smaller than the effect size of *r* = 0.67, as presumed in our a priori power calculations (see Methods for details). The estimated correlation is within the range of previous prediction markets on systematic replication projects with correlations of 0.42 in the RPP^10,15^, 0.30 in the EERP^16^ and 0.84 in the SSRP^17^, but we expected a stronger correlation because we selected studies with the highest and the lowest prices for replication. Consistent with the primary hypothesis test, there is also suggestive evidence of a difference in the replication rate between the ‘top-12’ (10 of 12) and ‘bottom-12’ (4 of 12) in our secondary hypothesis test. The difference of 50 percentage points is also reflected in the difference between the forecasted replication rates of 86.6% (‘top-12’) versus 29.6% (‘bottom-12’) in the decision market. However, the small sample size suggests caution against drawing firm conclusions about whether decision markets are appropriate for selecting studies for replication.

The pooled evidence from previous prediction market studies on replication outcomes suggests that markets are somewhat more accurate than surveys^45^, although the difference tends to be small. These indications are consistent with our results, yielding suggestive evidence of higher accuracy in terms of the absolute prediction error but not in terms of the squared prediction error (although, as noted above, failing to reject the null hypothesis for the squared prediction error does not imply that the null hypothesis is true). The estimated correlation between the average survey beliefs and the replication outcomes was almost as high for the survey as the prediction market (0.476 versus 0.505). The decision market prices and survey beliefs are also highly correlated with each other (*r* = 0.9). Since surveys are less resource intensive, simple polls can be an expedient alternative to decision markets for selecting which studies to replicate, even if they should be somewhat less accurate. Another potential method for selecting which studies to replicate would be to rely on the original *P* value for studies reporting statistically significant results^45^. Although the prediction accuracy appears to be somewhat lower for original *P* values than market and survey forecasts^45^, relying on *P* values may well be considered a practical alternative as it does not involve any additional data collection. Another possibility would be to use predicted replication probabilities from machine learning models to select studies for replication. There has been some progress in developing such models^120–123^, but evidence on whether they outperform markets or surveys is yet missing. Other potential mechanisms for selecting which studies to replicate include relying on general or study-specific characteristics (for example, connection to theory, surprise factor, sample size, effect size and importance)^25–28^, relying on cost–benefit considerations^29,30^, using Bayesian strategies^31,32^, determining the ‘replication value’^33^, adopting empirical audit and review^34^, selecting studies randomly^35^ or using predictions from laypeople^36,37^.

Using decision markets to select the studies with the highest and lowest predicted probabilities for replication is just one of the many potential selection rules for this methodology. Our goal was to test whether a decision market could distinguish findings that would replicate or not, and we aimed to maximize the statistical power of detecting an association between market prices and replication outcomes. For the practical application of decision markets, the choice of the selection mechanism will largely depend on the objective function. One selection rule would be to choose the studies with the highest predicted false positive likelihood, that is, the studies with the smallest market prices (in addition to at least one randomly selected study to ensure incentive compatibility). This decision mechanism would align with the objective of identifying and correcting false discoveries in the literature to facilitate an efficient allocation of resources for follow-up investigations. Another selection rule would be to replicate the studies with market predictions close to 50%, which reflects the highest possible uncertainty or disagreement regarding the likelihood of the original finding being genuinely true. Providing additional evidence on these claims could maximize the information value of replication studies, as well-powered replications will move the probability that the tested hypothesis is genuinely true towards 0% or 100%.

For our second primary hypothesis, we found strong evidence that original effect sizes are inflated on average compared with replication effect sizes, with a relative estimated average effect size of 45%. This is comparable to previous systematic replication studies, with relative average effect size estimates of 49% in the RPP^15^, 59% in the EERP^14^ and 54% in the SSRP^17^. The replication rate of 54% based on the statistical significance indicator is also similar to previous replication studies, with 36% in the RPP^15^, 61% in the EERP^14^ and 62% in the SSRP^17^. Caution should be exercised when comparing the replication results across these studies: the number of replications in each of the projects is small, only one focal result per paper has been selected for replication, and the particular journals and time periods considered differ. However, the results of all these studies are consistent with a replication rate of about 50% for both the binary statistical significance indicator and the continuous relative effect size indicator; compatible replication results have also been observed in the Many Labs replication projects^12–14^.

The ability of the statistical significance indicator to discriminate between true positives and false positives depends on replication power, and the relative average effect size of the studies that failed to replicate should be close to zero if the systematic replication study successfully separates false positives from true positives. The relative average estimated effect size of the 12 studies that failed to replicate according to the statistical significance indicator was 3.2%, which is close to zero and consistent with a successful separation between true positives and false positives. But also true positive findings can be expected to have exaggerated effect sizes in the published literature owing to a lack of statistical power^18,19^. In line with this, we found an estimated average effect size of 69.5% for the 14 studies that were successfully replicated based on the statistical significance indicator. These findings are consistent with similar analyses in the SSRP^17^ in which the estimated mean relative effect size among the studies that failed to replicate according to the statistical significance indicator was 0.3%, and the estimated mean relative effect size among the studies that replicated successfully was 73.1%. This illustrates how the combination of statistical significance and relative effect size can contribute to revealing possible false positives and true positives with exaggerated effect sizes.

Previous systematic replication studies have focused on laboratory experiments rather than online experiments. Concerns have been raised over data quality in online data collections using ‘crowd workers’, as via MTurk^88–94^, and part of the rationale for zeroing in on experiments conducted via MTurk was that we tend to share these concerns. However, the results of this study do not suggest that replicability is substantively lower for experiments conducted via MTurk compared with experiments conducted in physical laboratories for studies published in top journals; more evidence is needed to draw strong conclusions. Relatedly, the predicted average replicability rate of 57.6% in the decision market—despite widespread concerns about data quality on MTurk—is within the range of replication rate forecasts in previous prediction markets of 56% in the RPP^10^, 75% in the EERP^16^ and 63% in the SSRP^17^. We used IP quality checks^90,124^ to minimize the chances of low-quality participant data (see Methods for details), screening out participants before the random assignment into treatments. In total, across all 26 replications, 29% of the participants who accepted a ‘human intelligence task’ (HIT) failed the IP check and were excluded (this descriptive result was not preregistered; see Methods for further details). The replication results from our study should thus not be extrapolated to MTurk experiments not using a comparable screening procedure. An important caveat is that although our IP quality checks seem to have been effective in filtering out bots, this may not be the case for artificial responses generated by large language models such as ChatGPT, which could pose a challenge for collecting data online via platforms such as MTurk^125^.

There are several important limitations to our study. A successful replication, on its own, does not provide valid evidence for the tested hypothesis. It goes without saying that inference in replication studies is subject to type-I and type-II errors, just as in original studies. Moreover, a finding can be replicable while being based on an invalid experimental design, leading to biased results. An example of this would be an experimental design that systematically results in more attrition in one experimental treatment, causing selection bias in favour of the tested conjecture^88^. Likewise, a failed replication, on its own, does not provide direct evidence against the tested hypothesis. A finding can be unreplicable and based on an invalid experimental design, leaving the hypothesis untested. Although the replication rate for online experiments in our study appears to be similar to previous laboratory evidence, it does not necessarily imply that online and laboratory experiments provide equally valid evidence of the tested hypotheses.

Another limitation is that we only replicate a single focal result per paper, and the replication outcome does not necessarily generalize to other results reported in the original articles. Furthermore, we only gathered data from one online population using the same experimental design as in the original study. It cannot be ruled out that the difference in timing between the replication studies and the original studies has affected the replication results as a consequence of changes in the composition of the MTurk subject pool or the tested phenomenon having changed over time. Large-scale, multi-site replication studies that collect data across various populations and settings, similar to the Many Labs replication projects^12–14^, qualify as a promising method to shed light on the heterogeneity of replication effect sizes across populations and designs^126–128^ in future replication work, potentially increasing the strength of evidence for whether the hypothesis supported in the original study is likely true or not. Collaborative networks such as the Psychological Science Accelerator^129^ facilitate multi-site replication studies and can be a door opener to large and diverse samples.

Another caveat in interpreting our results is the lack of agreement about how to define and measure replicability. We chose to report the results for a broad set of replication indicators proposed in the literature and leave it to readers to gauge the strengths and weaknesses of the various measures. Decision markets come with the limitation of being a relatively resource-intensive tool, rendering simple polls an appealing alternative.

In our proof-of-concept investigation of using decision markets to assess replicability, decision markets show potential as a tool for selecting studies for replications, but further work is needed to draw strong conclusions. The observed replication rate of social science experiments based on data collections via MTurk published in PNAS is comparable to previous systematic replication projects of experimental studies in the social sciences, primarily based on lab experiments. However, the sample size of 26 replication studies is small, implying substantial uncertainty about both the estimated replication rate and the estimated association between the decision market prices and the replication rate. Our study is also limited to one scientific journal and may not be representative of social science results based on MTurk samples published in other journals, or studies using other online platforms for the data collection. Thus, prudence should be exercised in generalizing our findings and comparing replication results across studies.

## Methods

We preregistered an analysis plan for the project at OSF on 7 October 2021 before starting the survey data collection (that preceded the decision market and replications), which detailed the design of the study and the exact analyses for all planned analyses and tests (https://osf.io/xsp6g). Unless explicitly mentioned in the main text, we adhere exactly to our pre-analysis plan (PAP). The information in this section follows the PAP (with some of the information from the PAP reported in Supplementary Notes). Note that previous systematic replication projects such as the RPP^15^, the EERP^16^ and the SSRP^17^ did not file preregistrations of the overall study protocol and planned analyses.

Before starting the survey data collection, we also preregistered an analysis plan (‘replication report’) for each of the 41 potential replications included in the decision market at OSF after obtaining feedback from the original authors (https://osf.io/sejyp). After the replications had been conducted, the 26 replication reports of the replications selected for replication by the decision market were updated with the results of the replications and posted in the same OSF repository. Any deviations from the preregistered analysis plans for the 26 replications are detailed in the 26 ‘post-replication reports’ and listed in Supplementary Notes. We provided all original authors the opportunity to comment on the replication results (without a particular due date) and make the comments publicly available as we receive them alongside the post-replication reports on OSF (https://osf.io/sejyp).

Below, we provide further details on the inclusion criteria, the decision market set-up and the survey, the replications and the replication rate indicators included in the study. The preregistered analyses and tests were divided into descriptive results of the replication rate among the 26 replicated studies and hypothesis tests. The preregistered descriptive results were furthermore divided into primary replication indicators and secondary replication indicators, and the preregistered hypothesis tests were divided into (1) primary hypotheses, (2) secondary hypotheses and (3) exploratory analyses. See Supplementary Notes for more details about the preregistered hypothesis tests and exploratory analyses. We sought ethical approval from the Swedish Ethical Review Authority who had no ethical objections to the decision market part of the project and judged the replication part of the project to not be covered by the Swedish ethical review law (Dnr 2019-06501).

### Inclusion criteria for studies

We reviewed all PNAS articles from 2015 to 2018 and searched for the terms Amazon Mechanical Turk, MTurk and Turk. When we began planning our study at the start of 2019, we started reviewing the most recent articles published in PNAS. We then continued to look back in time, year by year, until we reached a sufficiently large number of studies to run a decision market. However, data collection was delayed after some original authors expressed concerns that the Covid-19 pandemic could affect the replication outcomes. We included all social sciences articles that fulfilled our inclusion criteria for (1) the journal and time period, (2) the platform on which the experiment was performed (MTurk), (3) the type of design (between-subjects or within-subject treatment design), (4) the equipment and materials needed to implement the experiment (the experiment had to be logistically feasible for us to implement) and (5) the results reported in the experiment (that there is at least one statistically significant *P* < 0.05 main or interaction effect in the main text). On the basis of the inclusion criteria, we identified 44 articles. After contacting the original authors, we ended up with 41 articles (the three excluded articles^130–132^ involved either software or platforms that no longer existed or methods we were unfamiliar with). In these 41 articles, we identified at least one critical finding that we could replicate. In cases where several studies in the same article fit the inclusion criteria, we randomly picked one of the studies; this was the case for 17 of the 26 replicated studies (Ames and Fiske^46^, Atir and Ferguson^47^, Baldwin and Lammers^48^, Boswell et al.^50^, Cooney et al.^56^, Genschow et al.^59^, Gheorghiu et al.^60^, Halevy and Halali^62^, Hofstetter et al.^65^, John et al.^69^, Jordan et al.^70^, Klein and O’Brien^73^, Kouchaki and Gino^74^, McCall et al.^76^, Rai et al.^81^, Stern et al.^84^ and Williams et al.^86^). In cases where the (randomly picked) study contained several conditions, we randomly picked which to compare to the control condition. After that, we looked for the central result with *P* < 0.05 for that particular study. If there were several statistically significant results, one was selected at random. The replication results thus only pertain to the single central result selected per paper, and the replication outcome does not necessarily generalize to other results reported in the original articles. For convenience, we refer to the replications as ‘replication of [study reference]’ though.

For Cheon and Hong^54^, the result chosen for replication is reported as part of a 2 × 2 ANOVA in the original article; since the paper does not report the main effect, the original authors kindly provided us with the corresponding estimates. For Gheorghiu et al.^60^, the result to be replicated is only reported with its *P* value in the paper; a precise estimate of the test statistic has been obtained from a re-analysis of the original data, which the original authors kindly provided. For the study by Kraus et al.^75^, we could not reproduce the result reported in the original article using the original data. The original authors acknowledged that there had been a reporting error in the original article. For the replication, we use the analysis described in the paper; the effect size and the test statistic reported in the original paper were replaced by the re-estimated result. For the study by Williams et al.^86^, the focal hypothesis test in the replication is based on a composite score of five suites of behaviour (which are tested separately in the original article) to have a single test. The original authors also report tests on composite measures in the Supplementary Information of their article, and they approved the choice to investigate the replicability of the focal hypothesis using a composite score. These changes are transparently reported in the replication reports for each study (see https://osf.io/sejyp for details).

### Decision market and prediction survey

We invited researchers to voluntarily participate in the decision market through public mailing lists (ESA and JDM lists) and social media (for example, Twitter/X); we also emailed colleagues asking them to distribute the call to participants within their professional networks. Participants were required to hold a PhD degree or to be a PhD student currently. In the decision market, participants bet on whether or not the specific result chosen for each study would replicate based on the statistical significance indicator (*P* < 0.05 in the replication and an effect in the same direction as in the original study) as a criterion for replication (thus a binary outcome, as discussed below). Before the decision market, participants filled out a survey where we asked them to assign a probability of successful replication to each of the 41 results. The survey is available at https://osf.io/a24zq. Completing the survey was a prerequisite for participating in the markets. We started the recruitment of participants for the decision market on 4 October (2021), and we started sending out the prediction survey on 8 October to those who had signed up for the study (participants who signed up after 8 October received the survey invitation a few days after their registration). The deadline for registering as a participant was 29 October, and the deadline for completing the survey was 5 November. Overall, 289 participants signed up to participate and were forwarded the link to the survey; 193 participants started the survey, and 162 completed it by the due date. The forecasters were from the following fields of research: psychology (37.7%), economics (34.6%), management (7.4%), political science (4.9%), sociology (1.9%) and other fields (13.6%). No additional demographic information was collected.

In the survey, we asked participants to assess, for each replication study, (1) the likelihood that the hypothesis will successfully replicate (on a scale from 0% to 100%); (2) their stated expertise for the study/the hypothesis (on a scale from 1 to 7); and (3) whether they believe that the pandemic has affected the likelihood of replication. The question about the pandemic was measured on a scale from −3 (‘the pandemic has definitely decreased the probability of replication’) to 3 (‘the pandemic has definitely increased the probability of replication’); the 0 midpoint implies that they do not think that the pandemic has affected the probability of replication. The survey was not incentivized.

The decision market opened on 8 November (2021) and closed after 2 weeks on 22 November (and before the decision market opened, participants had at least 1 week to complete the prediction survey). In the decision market, participants could trade (bet) on whether they expected the 41 studies to replicate. While participants had the opportunity to bet on the replication outcome of the 41 studies, we did not carry out replications for all 41 studies, but the final decision market prices determined which studies to replicate. We replicated the 12 studies that had the highest and the 12 studies that had the lowest market prices when the market closed. In addition, 2 out of the remaining 17 studies were randomly selected for replication to ensure a non-zero probability for each study to be replicated (that is, we replicated 12 + 12 + 2 = 26 studies in total). Since payoffs are only determined based on forecasts of studies that were eventually replicated, payoffs were scaled up by the inverse of their probability of being selected for replication in the decision rule (see below for details). This incentive scheme encourages trading based on traders’ true beliefs, even though some studies will not be replicated. Consequently, participants have the incentive to buy shares of a particular study whenever they believe that the likelihood of replication is higher than the current market price; likewise, participants have an incentive to (short) sell shares whenever they believe that the likelihood of replication is lower than the current market price. Thus, as long as the market price differs from the predicted likelihood of replication for a participant, the participant has an incentive to buy or (short) sell shares of a particular study and realizing a trade according to a trader’s belief will move the market price in the direction of the trader’s belief. The decision rule for which studies to replicate was based on final market prices and was common knowledge to the market participants; the instructions (provided to participants who completed the prediction survey by the due date) are available at https://osf.io/a24zq/.

We chose 12 studies with the lowest predicted probability and 12 studies with the highest predicted probability based on a power calculation using the pooled data from our previous prediction market studies^45^. The power calculations were conducted by randomly sampling 41 studies from the dataset described in Gordon et al.^45^ in a simulation with 10,000 iterations and then selecting the forecasts and outcomes from the 12 studies with the lowest predicted probability, the 12 studies with the highest predicted probability and 2 random studies. We failed to set a random seed for the simulation when the study was conducted, implying that the preregistered power estimates could not be numerically reproduced when we wrote up the study results. For full transparency, we report the power estimates included in the PAP in parentheses below. The median point-biserial correlation coefficient across the 10,000 runs is 0.671 (reported as 0.66 in the PAP), and we have 91.0% power (reported as >90% in the PAP) to detect a statistically significant correlation (*n* = 26) between the decision market prices and the replication outcomes at the 0.5% level and 99.4% power (reported as >95% in the PAP) to detect a statistically significant correlation at the 5% level, which is our first primary hypothesis test. As a secondary hypothesis test, we test if the fraction of studies that successfully replicate differs between the 12 studies with the highest and the 12 studies with the lowest predicted replication probabilities using Fisher’s exact test. Applying the same sampling approach as for primary hypothesis 1, the median difference in replication rates between the 12 studies with the highest and the 12 studies with the lowest market prices is 0.663; the secondary test (*n* = 24) has 66.5% power at the 0.5% level (reported as 66% in the PAP) and 94.9% power at the 5% level (reported as 95% in the PAP). The code for the power simulations is available at https://osf.io/47drs.

#### Implementation of the decision market

We used a web-based trading platform, similar to the ones used in Camerer et al.^16,17^ and identical to the one used in Botvinik-Nezer et al.^6^. The trading platform involves two main views: (1) the market overview and (2) the trading page. The market overview listed the 41 assets (that is, one corresponding to each study) in tabular format, including information on the current price for buying a share and the number of shares held (separated for long and short positions). Via the trading page, which was shown after clicking on any of the assets, participants could make investment decisions (that is, buy or sell shares) and view price developments in graphical format for the particular asset.

#### Trading and incentivization

Decision market participants received an endowment of 100 tokens corresponding to USD 50. Once the markets opened, market participants could use the tokens to trade shares of the assets available in the market. An automated market maker, implementing a logarithmic market scoring rule^133^, determined the assets’ prices. At the beginning of the markets, all assets were valued at 0.50 tokens per share. The market maker calculated the share price for each infinitesimal transaction and updated the price based on the scoring rule. With this mechanism, participants had incentives to invest according to their beliefs^43,44^. With the logarithmic scoring rule, the price *p* for an infinitesimal trade is determined as *p* = e^s/b^ ÷ (e^s/b^ + 1), where s denotes the net sales (shares held − shares borrowed) that the market maker has done so far in a market; the liquidity parameter b determines how strongly the market price is affected by trade and was set to *b* = 100, implying that by investing ten tokens, traders could move the price of a single asset from 0.50 to about 0.55. We opted for the same value of b as the one used in the prediction markets in the SSRP^17^, which appears intuitively sensible in terms of striking a good balance between price sensitivity and liquidity. Notwithstanding, it is worth noting that it is unclear whether or not our results are sensitive to the choice of this parameter. Decision market participants were paid only for studies chosen for replication (based on their final holdings). Participants received one token per correct share for the replications with the 12 lowest and 12 highest final market prices. For the two randomly selected replications, participants received 17 ÷ 2 = 8.5 tokens for each share; for replications that were not chosen for replication, participants received no compensation for their holdings. We followed this procedure to keep information revelation in the decision market incentive compatible, with the increased payouts for the randomly selected studies compensating for the ‘voided’ shares in studies not chosen for replication. Participants were paid after all 26 replications had been completed.

#### Participation

A total of 193 participants completed the prediction survey (a prerequisite to participate in market trading) after providing consent to participate and were subsequently invited to trade on the decision market. Of these 193 participants, 162 (83.9%) traded in the market at least once. During the 2-week trading period, a total of 4,412 transactions were recorded. On average, each trader prompted 27.2 transactions (s.d. = 30.7; min = 1, max = 185). The average number of traders per hypothesis was 65.1 (s.d. = 15.3; min = 35, max = 98); the average number of transactions recorded per hypothesis was 107.6 (s.d. = 35.2; min = 56, max = 213). See Supplementary Table 2 for descriptive statistics on the trading activity for each market.

### Replications

We carry out close replications^107^ as closely as possible following the experimental design, sample restrictions, exclusion criteria and analysis as used in the original studies and carried out in the same population (Amazon Mechanical Turk) as the original studies. The replications started in January 2022 and were completed in October 2023. The replications were planned and preregistered by five replication teams: a team at CalTech, LMU and Wharton; a team at the Stockholm School of Economics; a team at the National University of Singapore; a team at the University of Amsterdam; and a team at the University of Innsbruck.

#### Participants in replication studies

All replications were carried out at Amazon Mechanical Turk as in the original studies. We ensured that participants could only participate once using the same account in a specific study. If the original study had not specified an HIT approval rate, we recruited participants with an HIT approval rate of at least 95%; if the original study had specified a higher approval rate, we applied the same requirement as used in the original study.

To ward off concerns about impaired data quality owing to low-attention participants and bots^88–94^, we implemented several ‘quality filters’. Particularly, before redirecting participants to each study, we forwarded the IP addresses to https://www.ipqualityscore.com/ for a quality check to minimize the chances of low-quality participant data (we initially planned to use this filter ex post, but during the data collection of the first two replication studies of Klein and O’Brien^73^ and Halevy and Halali^62^, we decided to set it up so that the IP address quality check happened before participants got redirected to the study). Participants for whom one or more of the following was true could not proceed with participating in the study: fraud score ≥ 85; TOR = true; VPN = true; bot = true; abuse velocity = high. This means that, for example, participants were not allowed to use a virtual private network (VPN) or Tor connections or participate if they had IP addresses that had recently engaged in automated bot activity (the VPN exclusions were made ex ante, that is, before participants were redirected to the study, for 4 studies and ex post for 22 studies). After that, in all replications, participants were first shown a Captcha and then provided informed consent. After this, we included an attention check that participants had to pass to proceed to the study (with the exception of Reeck et al.^82^; see Supplementary Section 4 for details). The attention check was implemented in addition to any other potential attention check(s) used in the original study. All these exclusions based on the ‘quality filters’ were preregistered, but the PAP did not specify if participants would be excluded before or after participating in the study.

The individual replication studies sometimes also used additional exclusion criteria that are detailed in the preregistered replication report for each replication (we tried to use the same exclusion criteria for the replications as used in the original studies as much as possible). The replication sample sizes defined below are the sample sizes after any exclusions of participants.

#### Replication sample sizes

The replications were carried out with high statistical power. Replication sample sizes were based on having 90% power to detect 2/3 of the effect size reported in the original study (with the effect size converted to Cohen’s *d* to have a common standardized effect size measure across the original studies and the replication studies). See Supplementary Notes for more details about the power calculations and replication sample sizes. The criteria for replication were an effect in the same direction as the original study and a *P* value < 0.05 (in a two-sided test). In cases where this power estimation led to a sample size smaller than the original one, we used the same sample size as in the original study. The average replication sample (*n̅* = 1,018) size was 3.5 times as large as the average sample size of the original studies (*n̅* = 292). We continued the data collection for each replication until we reached at least the preregistered sample size after exclusions for that replication, and this led to slightly larger replication sample sizes than preregistered in all replications except one (as it is not possible with exclusion criteria to get an exact sample size as the number of exclusions is not known ex ante).

#### Conversion of effect sizes to Cohen’s *d*

We converted the effect sizes of all the original studies and all the replication studies to Cohen’s *d* to have a standardized effect size (the effect size in the original study was always assigned a positive sign; the effect size in the replication study was assigned a positive sign if the effect was in the same direction as in the original study and a negative sign if the effect was in the opposite direction of the original study). See Supplementary Notes for details about the conversion of effect sizes to Cohen’s *d*.

#### Replication reports

For each of the 41 studies, we prepared a pre-replication plan/report stating the hypothesis we had chosen from each paper and how we planned to proceed with the replication study. These reports were shared with the original authors for feedback, and at least one original author from each paper replied. These pre-replication reports were posted at OSF (https://osf.io/sejyp) at the same time as the PAP and before the start of the prediction survey (that preceded the decision markets and the replication data collections). For those studies that were selected for replication, we have updated the replication reports with the replication results after the replications were completed. After sharing them with the original authors for feedback, we have posted the updated replication reports at OSF as well (https://osf.io/sejyp). In addition, we reached out to the original authors for their comments on the replication reports and results. We promised to make their comments available along with the replication reports, and any comments received can be found at https://osf.io/sejyp.

#### Incentivization in the replication experiments

We standardized payments across all replications such that studies had a certain show-up fee depending on the expected length of the study. In particular, we paid an hourly fee of USD 8.00 for all studies, and we calculated the show-up fee for each study based on the expected length of the study. For all studies, we implemented a minimum payoff of USD 1.00. For studies with incentive payments, we used the same incentive payment as in the original study, paid in addition to the show-up fee. If we faced problems in recruiting participants, we increased the show-up fee, which happened for two studies^61,65^.

### Replication indicators

#### Statistical significance criterion (primary indicator)

The first primary replication indicator was the statistical significance criterion—that is, whether the replication resulted in an effect size in the same direction as the original study and a two-sided *P* value less than 0.05. Unless otherwise stated above, we used the same statistical test as in the original study. We report the replication rate (that is, the fraction of the 26 studies that replicated according to this criterion) and the 95% Clopper–Pearson CI of this fraction in ‘Results’. We also report the 95% CI of the replication effect size for each of the 26 replication studies in Fig. 2 and Supplementary Table 3.

#### Relative effect sizes (primary indicator)

As a second primary replication indicator, we used relative effect sizes. Relative effect sizes were estimated in two different ways. We report the mean effect size of all 26 replications and compare it to the mean effect size of the 26 original studies (see also primary hypothesis test 2 below). We furthermore estimate the relative effect size of each replication (the replication effect size divided by the original effect size) and estimate the mean of this variable for the 26 replication studies and the 95% CI of this mean (based on a one-sample *t*-test). We report both of these measures of the relative effect size separately for the replications that replicate and those that do not. These results are reported in ‘Results’, Fig. 3 and Supplementary Table 3.

#### Small-telescopes approach (secondary indicator)

We also used the small-telescopes approach^112^. For this indicator, we estimated whether the replication effect size was significantly smaller (using a one-sided test at the 5% level) than a ‘small effect’, defined as the effect size the original study would have had 33% power to detect. For studies using *t*-tests (or *F*-tests converted to a *t*-test statistic), we based ‘the small effect size’ on the effect size that a *t*-test had 33% power to detect (at the 5% level in a two-sided test); for studies using *z*-test statistics (or chi-square tests converted to a *z*-test statistic), we based ‘the small effect size’ on the effect size that a *z*-test had 33% power to detect (at the 5% level in a two-sided test). To test whether the replication effect size was significantly smaller than ‘the small effect size’ in a one-sided test at the 5% level, we estimated a 90% CI of the replication effect size. We tested if the 90% CI overlapped the small effect size with CIs constructed as described in Supplementary Notes. If the effect size in the replication was significantly smaller than this ‘small effect size’, the result was considered a failed replication; otherwise, it was considered successful. We report the fraction of studies that replicate according to this criterion and the 95% Clopper–Pearson CI of this fraction. The small-telescopes results are reported in Fig. 4 and Supplementary Table 4.

#### Bayes factors (secondary indicators)

We also compute the one-sided default Bayes factors on the replication data, allowing us to obtain the strength of evidence in favour of the hypothesis that stipulates an effect in the direction of the original experiment (where a default prior in terms of a truncated Cauchy distribution with scale 0.707 was assigned to the size of the effect) versus the null hypothesis that stipulates the effect to be absent^113^. In addition, we also computed (one-sided) replication Bayes factors, which quantifies the additional evidence for the hypothesis given the evidence already provided by the original study^114^. (We are counting the one-sided default and replication as Bayes factors as two separate indicators, which they are.) These results are reported in Fig. 5 and Supplementary Table 4. We use the evidence categories proposed by Jeffreys^115^ to interpret the Bayes factors. A detailed report on the estimation of the Bayes factors is available at https://osf.io/47drs/.

#### Meta-analytic effect sizes (secondary indicator)

We estimated the meta-analytic estimate of the effect size by combining the original result and the replication result in a fixed-effect meta-analysis. We report the fraction of the 26 studies that replicated according to the 0.05 and the 0.005 significance threshold and the 95% Clopper–Pearson CI of these fractions. We also use the stricter 0.005 significance threshold as a replication indicator for the meta-analytic effect sizes because this is similar to observing two studies (an original study and a replication study) that are significant at the 0.05 level. We report these results in Results, Fig. 6 and Supplementary Table 4.

### Reporting summary

Further information on research design is available in the Nature Portfolio Reporting Summary linked to this article.

## Supplementary information

Supplementary Information Supplementary Notes, references, Fig. 1 and Tables 1–8.

Reporting Summary

## Data Availability

The data reported in this paper are tabulated in Supplementary Tables 1–8. The replication reports (both the pre-replication and the post-replication versions), the pre-analysis plan, the data from the survey and the decision market, and the data for each of the 26 replications are available at the project’s OSF repository (https://osf.io/sk82q).

## Code availability

The analysis scripts, generating all results, figures and tables reported in the main text and the Supplementary Information, are available at the project’s OSF repository (https://osf.io/sk82q).

## References

[1] Ioannidis, J. P. A. Why most published research findings are false. *PLoS Med.* **2**, e124 (2005). 16060722 10.1371/journal.pmed.0020124 PMC1182327

[2] Leamer, E. E. Let’s take the con out of econometrics. *Am. Econ. Rev.* **73**, 31–43 (1983).

[3] Begley, C. G. & Ellis, L. M. Raise standards for preclinical cancer research. *Nature* **483**, 531–533 (2012). 22460880 10.1038/483531a

[4] McNutt, M. Reproducibility. *Science* **343**, 229–229 (2014). 24436391 10.1126/science.1250475

[5] Gertler, P., Galiani, S. & Romero, M. How to make replication the norm. *Nature* **554**, 417–419 (2018). 32094957 10.1038/d41586-018-02108-9

[6] Botvinik-Nezer, R. et al. Variability in the analysis of a single neuroimaging dataset by many teams. *Nature* **582**, 84–88 (2020). 32483374 10.1038/s41586-020-2314-9 PMC7771346

[7] Breznau, N. et al. Observing many researchers using the same data and hypothesis reveals a hidden universe of uncertainty. *Proc. Natl Acad. Sci. USA* **119**, e2203150119 (2022). 36306328 10.1073/pnas.2203150119 PMC9636921

[8] Delios, A. et al. Examining the generalizability of research findings from archival data. *Proc. Natl Acad. Sci. USA* **119**, e2120377119 (2022). 35858443 10.1073/pnas.2120377119 PMC9335312

[9] Huber, C. et al. Competition and moral behavior: a meta-analysis of forty-five crowd-sourced experimental designs. *Proc. Natl Acad. Sci. USA* **120**, e2215572120 (2023). 37252958 10.1073/pnas.2215572120 PMC10266008

[10] Dreber, A. et al. Using prediction markets to estimate the reproducibility of scientific research. *Proc. Natl Acad. Sci. USA* **112**, 15343–15347 (2015). 26553988 10.1073/pnas.1516179112 PMC4687569

[11] Maniadis, Z., Tufano, F. & List, J. A. To replicate or not to replicate? Exploring reproducibility in economics through the lens of a model and a pilot study. *Econ. J.* **127**, F209–F235 (2017).

[12] Klein, R. A. et al. Investigating variation in replicability: a ‘many labs’ replication project. *Soc. Psychol.* **45**, 142–152 (2014).

[13] Ebersole, C. R. et al. Many Labs 3: evaluating participant pool quality across the academic semester via replication. *J. Exp. Soc. Psychol.* **67**, 68–82 (2016).

[14] Klein, R. A. et al. Many Labs 2: investigating variation in replicability across samples and settings. *Adv. Methods Pract. Psychol. Sci.* **1**, 443–490 (2018).

[15] Open Science Collaboration Estimating the reproducibility of psychological science. *Science* **349**, aac4716 (2015). 26315443 10.1126/science.aac4716

[16] Camerer, C. F. et al. Evaluating replicability of laboratory experiments in economics. *Science* **351**, 1433–1436 (2016). 26940865 10.1126/science.aaf0918

[17] Camerer, C. F. et al. Evaluating the replicability of social science experiments in *Nature* and *Science* between 2010 and 2015. *Nat. Hum. Behav.* **2**, 637–644 (2018). 31346273 10.1038/s41562-018-0399-z

[18] Ioannidis, J. P. A. Why most discovered true associations are inflated. *Epidemiology* **19**, 640 (2008). 18633328 10.1097/EDE.0b013e31818131e7

[19] Button, K. S. et al. Power failure: why small sample size undermines the reliability of neuroscience. *Nat. Rev. Neurosci.* **14**, 365–376 (2013). 23571845 10.1038/nrn3475

[20] Wegener, D. T., Fabrigar, L. R., Pek, J. & Hoisington-Shaw, K. Evaluating research in personality and social psychology: considerations of statistical power and concerns about false findings. *Pers. Soc. Psychol. Bull.* **48**, 1105–1117 (2022). 34308722 10.1177/01461672211030811

[21] Simmons, J. P., Nelson, L. D. & Simonsohn, U. False-positive psychology: undisclosed flexibility in data collection and analysis allows presenting anything as significant. *Psychol. Sci.* **22**, 1359–1366 (2011). 22006061 10.1177/0956797611417632

[22] John, L. K., Loewenstein, G. & Prelec, D. Measuring the prevalence of questionable research practices with incentives for truth telling. *Psychol. Sci.* **23**, 524–532 (2012). 22508865 10.1177/0956797611430953

[23] Finkel, E. J., Eastwick, P. W. & Reis, H. T. Replicability and other features of a high-quality science: toward a balanced and empirical approach. *J. Pers. Soc. Psychol.* **113**, 244–253 (2017). 28714730 10.1037/pspi0000075

[24] Flake, J. K., Davidson, I. J., Wong, O. & Pek, J. Construct validity and the validity of replication studies: a systematic review. *Am. Psychol.* **77**, 576–588 (2022). 35482669 10.1037/amp0001006

[25] Pittelkow, M.-M. et al. The process of replication target selection in psychology: what to consider? *R. Soc. Open Sci.* **10**, 210586 (2023). 36756069 10.1098/rsos.210586 PMC9890109

[26] Makel, M. C., Plucker, J. A. & Hegarty, B. Replications in psychology research: how often do they really occur? *Perspect. Psychol. Sci.* **7**, 537–542 (2012). 26168110 10.1177/1745691612460688

[27] Lindsay, D. S. Replication in psychological science. *Psychol. Sci.* **26**, 1827–1832 (2015). 26553013 10.1177/0956797615616374

[28] Block, J. & Kuckertz, A. Seven principles of effective replication studies: strengthening the evidence base of management research. *Manag. Rev. Q.* **68**, 355–359 (2018).

[29] Coles, N. A., Tiokhin, L., Scheel, A. M., Isager, P. M. & Lakens, D. The costs and benefits of replication studies. *Behav. Brain Sci.* **41**, e124 (2018). 31064512 10.1017/S0140525X18000596

[30] Alipourfard, N. et al. Systematizing confidence in open research and evidence (SCORE). Preprint at *SocArXiv* https://doi.org/10/hn4g (2021).

[31] Hardwicke, T. E., Tessler, M. H., Peloquin, B. N. & Frank, M. C. A Bayesian decision-making framework for replication. *Behav. Brain Sci.* **41**, e132 (2018). 31064517 10.1017/S0140525X18000675

[32] Field, S. M., Hoekstra, R., Bringmann, L. & van Ravenzwaaij, D. When and why to replicate: as easy as 1, 2, 3? *Collabra Psychol.* **5**, 46 (2019).

[33] Isager, P. M. et al. Deciding what to replicate: a decision model for replication study selection under resource and knowledge constraints. *Psychol. Methods* **28**, 438–451 (2023). 34928679 10.1037/met0000438

[34] O’Donnell, M. et al. Empirical audit and review and an assessment of evidentiary value in research on the psychological consequences of scarcity. *Proc. Natl Acad. Sci. USA* **118**, e2103313118 (2021). 34711679 10.1073/pnas.2103313118 PMC8612349

[35] Kuehberger, A. & Schulte-Mecklenbeck, M. Selecting target papers for replication. *Behav. Brain Sci.* **41**, e139 (2018). 31064529 10.1017/S0140525X18000742

[36] Hoogeveen, S., Sarafoglou, A. & Wagenmakers, E.-J. Laypeople can predict which social-science studies will be replicated successfully. *Adv. Methods Pract. Psychol. Sci.* **3**, 267–285 (2020).

[37] Marcoci, A. et al. Predicting the replicability of social and behavioural science claims from the COVID-19 Preprint Replication Project with structured expert and novice groups. Preprint at *MetaArXiv*10.31222/osf.io/xdsjf (2023).

[38] Wolfers, J. & Zitzewitz, E. Prediction markets. *J. Econ. Perspect.* **18**, 107–126 (2004).

[39] Arrow, K. J. et al. The promise of prediction markets. *Science* **320**, 877–878 (2008). 18487176 10.1126/science.1157679

[40] Tziralis, G. & Tatsiopoulos, I. Prediction markets: an extended literature review. *J. Predict. Mark.* **1**, 75–91 (2012).

[41] Hanson, R. Decision markets. *IEEE Intell. Syst.* **14**, 16–19 (1999).

[42] Hanson, R. Combinatorial information market design. *Inf. Syst. Front.* **5**, 107–119 (2003).

[43] Chen, Y., Kash, I., Ruberry, M. & Shnayder, V. Decision markets with good incentives. In *Proc. Internet and Network Economics* (eds Chen, N. et al.) 72–83 (Springer, 2011).

[44] Wang, W. & Pfeiffer, T. Securities based decision markets. In *Proc. Distributed Artificial Intelligence* Vol. 13170 (eds Chen, J. et al.) 79–92 (Springer, 2022).

[45] Gordon, M., Viganola, D., Dreber, A., Johannesson, M. & Pfeiffer, T. Predicting replicability—analysis of survey and prediction market data from large-scale forecasting projects. *PLoS ONE* **16**, e0248780 (2021). 33852589 10.1371/journal.pone.0248780 PMC8046229

[46] Ames, D. L. & Fiske, S. T. Perceived intent motivates people to magnify observed harms. *Proc. Natl Acad. Sci. USA* **112**, 3599–3605 (2015). 25733850 10.1073/pnas.1501592112 PMC4378403

[47] Atir, S. & Ferguson, M. J. How gender determines the way we speak about professionals. *Proc. Natl Acad. Sci. USA* **115**, 7278–7283 (2018). 29941572 10.1073/pnas.1805284115 PMC6048538

[48] Baldwin, M. & Lammers, J. Past-focused environmental comparisons promote proenvironmental outcomes for conservatives. *Proc. Natl Acad. Sci. USA* **113**, 14953–14957 (2016). 27956619 10.1073/pnas.1610834113 PMC5206530

[49] Bear, A., Fortgang, R. G., Bronstein, M. V. & Cannon, T. D. Mistiming of thought and perception predicts delusionality. *Proc. Natl Acad. Sci. USA* **114**, 10791–10796 (2017). 28923963 10.1073/pnas.1711383114 PMC5635918

[50] Boswell, R. G., Sun, W., Suzuki, S. & Kober, H. Training in cognitive strategies reduces eating and improves food choice. *Proc. Natl Acad. Sci. USA* **115**, E11238–E11247 (2018). 30420496 10.1073/pnas.1717092115 PMC6275472

[51] Caruso, E. M., Burns, Z. C. & Converse, B. A. Slow motion increases perceived intent. *Proc. Natl Acad. Sci. USA* **113**, 9250–9255 (2016). 27482091 10.1073/pnas.1603865113 PMC4995977

[52] Casella, A., Kartik, N., Sanchez, L. & Turban, S. Communication in context: interpreting promises in an experiment on competition and trust. *Proc. Natl Acad. Sci. USA* **115**, 933–938 (2018). 29339524 10.1073/pnas.1714171115 PMC5798344

[53] Chao, M. Demotivating incentives and motivation crowding out in charitable giving. *Proc. Natl Acad. Sci. USA* **114**, 7301–7306 (2017). 28655844 10.1073/pnas.1616921114 PMC5514700

[54] Cheon, B. K. & Hong, Y.-Y. Mere experience of low subjective socioeconomic status stimulates appetite and food intake. *Proc. Natl Acad. Sci. USA* **114**, 72–77 (2017). 27994148 10.1073/pnas.1607330114 PMC5224403

[55] Clarkson, J. J. et al. The self-control consequences of political ideology. *Proc. Natl Acad. Sci. USA* **112**, 8250–8253 (2015). 26100890 10.1073/pnas.1503530112 PMC4500254

[56] Cooney, G., Gilbert, D. T. & Wilson, T. D. When fairness matters less than we expect. *Proc. Natl Acad. Sci. USA* **113**, 11168–11171 (2016). 27638203 10.1073/pnas.1606574113 PMC5056033

[57] Côté, S., House, J. & Willer, R. High economic inequality leads higher-income individuals to be less generous. *Proc. Natl Acad. Sci. USA* **112**, 15838–15843 (2015). 26598668 10.1073/pnas.1511536112 PMC4702979

[58] Flesch, T., Balaguer, J., Dekker, R., Nili, H. & Summerfield, C. Comparing continual task learning in minds and machines. *Proc. Natl Acad. Sci. USA* **115**, E10313–E10322 (2018). 30322916 10.1073/pnas.1800755115 PMC6217400

[59] Genschow, O., Rigoni, D. & Brass, M. Belief in free will affects causal attributions when judging others’ behavior. *Proc. Natl Acad. Sci. USA* **114**, 10071–10076 (2017). 28855342 10.1073/pnas.1701916114 PMC5617252

[60] Gheorghiu, A. I., Callan, M. J. & Skylark, W. J. Facial appearance affects science communication. *Proc. Natl Acad. Sci. USA* **114**, 5970–5975 (2017). 28533389 10.1073/pnas.1620542114 PMC5468637

[61] Guilbeault, D., Becker, J. & Centola, D. Social learning and partisan bias in the interpretation of climate trends. *Proc. Natl Acad. Sci. USA* **115**, 9714–9719 (2018). 30181271 10.1073/pnas.1722664115 PMC6166837

[62] Halevy, N. & Halali, E. Selfish third parties act as peacemakers by transforming conflicts and promoting cooperation. *Proc. Natl Acad. Sci. USA* **112**, 6937–6942 (2015). 26038546 10.1073/pnas.1505067112 PMC4460509

[63] Handley, I. M., Brown, E. R., Moss-Racusin, C. A. & Smith, J. L. Quality of evidence revealing subtle gender biases in science is in the eye of the beholder. *Proc. Natl Acad. Sci. USA* **112**, 13201–13206 (2015). 26460001 10.1073/pnas.1510649112 PMC4629390

[64] Hoffman, K. M., Trawalter, S., Axt, J. R. & Oliver, M. N. Racial bias in pain assessment and treatment recommendations, and false beliefs about biological differences between blacks and whites. *Proc. Natl Acad. Sci. USA* **113**, 4296–4301 (2016). 27044069 10.1073/pnas.1516047113 PMC4843483

[65] Hofstetter, R., Rüppell, R. & John, L. K. Temporary sharing prompts unrestrained disclosures that leave lasting negative impressions. *Proc. Natl Acad. Sci. USA* **114**, 11902–11907 (2017). 29078302 10.1073/pnas.1706913114 PMC5692543

[66] Horne, Z., Powell, D., Hummel, J. E. & Holyoak, K. J. Countering antivaccination attitudes. *Proc. Natl Acad. Sci. USA* **112**, 10321–10324 (2015). 26240325 10.1073/pnas.1504019112 PMC4547299

[67] Isley, S. C., Stern, P. C., Carmichael, S. P., Joseph, K. M. & Arent, D. J. Online purchasing creates opportunities to lower the life cycle carbon footprints of consumer products. *Proc. Natl Acad. Sci. USA* **113**, 9780–9785 (2016). 27528670 10.1073/pnas.1522211113 PMC5024622

[68] Jachimowicz, J. M., Chafik, S., Munrat, S., Prabhu, J. C. & Weber, E. U. Community trust reduces myopic decisions of low-income individuals. *Proc. Natl Acad. Sci. USA* **114**, 5401–5406 (2017). 28400516 10.1073/pnas.1617395114 PMC5448192

[69] John, L. K., Barasz, K. & Norton, M. I. Hiding personal information reveals the worst. *Proc. Natl Acad. Sci. USA* **113**, 954–959 (2016). 26755591 10.1073/pnas.1516868113 PMC4743808

[70] Jordan, J. J., Hoffman, M., Nowak, M. A. & Rand, D. G. Uncalculating cooperation is used to signal trustworthiness. *Proc. Natl Acad. Sci. USA* **113**, 8658–8663 (2016). 27439873 10.1073/pnas.1601280113 PMC4978259

[71] Jun, Y., Meng, R. & Johar, G. V. Perceived social presence reduces fact-checking. *Proc. Natl Acad. Sci. USA* **114**, 5976–5981 (2017). 28533396 10.1073/pnas.1700175114 PMC5468680

[72] KC, R. P., Kunter, M. & Mak, V. The influence of a competition on noncompetitors. *Proc. Natl Acad. Sci. USA* **115**, 2716–2721 (2018). 29483272 10.1073/pnas.1717301115 PMC5856535

[73] Klein, N. & O’Brien, E. People use less information than they think to make up their minds. *Proc. Natl Acad. Sci. USA* **115**, 13222–13227 (2018). 30530692 10.1073/pnas.1805327115 PMC6310859

[74] Kouchaki, M. & Gino, F. Memories of unethical actions become obfuscated over time. *Proc. Natl Acad. Sci. USA* **113**, 6166–6171 (2016). 27185941 10.1073/pnas.1523586113 PMC4896721

[75] Kraus, M. W., Rucker, J. M. & Richeson, J. A. Americans misperceive racial economic equality. *Proc. Natl Acad. Sci. USA* **114**, 10324–10331 (2017). 28923915 10.1073/pnas.1707719114 PMC5625917

[76] McCall, L., Burk, D., Laperrière, M. & Richeson, J. A. Exposure to rising inequality shapes Americans’ opportunity beliefs and policy support. *Proc. Natl Acad. Sci. USA* **114**, 9593–9598 (2017). 28831007 10.1073/pnas.1706253114 PMC5594671

[77] Morris, A., MacGlashan, J., Littman, M. L. & Cushman, F. Evolution of flexibility and rigidity in retaliatory punishment. *Proc. Natl Acad. Sci. USA* **114**, 10396–10401 (2017). 28893996 10.1073/pnas.1704032114 PMC5625901

[78] Mummolo, J. Militarization fails to enhance police safety or reduce crime but may harm police reputation. *Proc. Natl Acad. Sci. USA* **115**, 9181–9186 (2018). 30126997 10.1073/pnas.1805161115 PMC6140536

[79] Payne, B. K., Brown-Iannuzzi, J. L. & Hannay, J. W. Economic inequality increases risk taking. *Proc. Natl Acad. Sci. USA* **114**, 4643–4648 (2017). 28416655 10.1073/pnas.1616453114 PMC5422783

[80] Phillips, J. & Cushman, F. Morality constrains the default representation of what is possible. *Proc. Natl Acad. Sci. USA* **114**, 4649–4654 (2017). 28420792 10.1073/pnas.1619717114 PMC5422784

[81] Rai, T. S., Valdesolo, P. & Graham, J. Dehumanization increases instrumental violence, but not moral violence. *Proc. Natl Acad. Sci. USA* **114**, 8511–8516 (2017). 28739935 10.1073/pnas.1705238114 PMC5559031

[82] Reeck, C., Wall, D. & Johnson, E. J. Search predicts and changes patience in intertemporal choice. *Proc. Natl Acad. Sci. USA* **114**, 11890–11895 (2017). 29078303 10.1073/pnas.1707040114 PMC5692544

[83] Schilke, O., Reimann, M. & Cook, K. S. Power decreases trust in social exchange. *Proc. Natl Acad. Sci. USA* **112**, 12950–12955 (2015). 26438869 10.1073/pnas.1517057112 PMC4620861

[84] Stern, C., West, T. V. & Rule, N. O. Conservatives negatively evaluate counterstereotypical people to maintain a sense of certainty. *Proc. Natl Acad. Sci. USA* **112**, 15337–15342 (2015). 26621712 10.1073/pnas.1517662112 PMC4687596

[85] Vacharkulksemsuk, T. et al. Dominant, open nonverbal displays are attractive at zero-acquaintance. *Proc. Natl Acad. Sci. USA* **113**, 4009–4014 (2016). 27035937 10.1073/pnas.1508932113 PMC4839399

[86] Williams, K. E. G., Sng, O. & Neuberg, S. L. Ecology-driven stereotypes override race stereotypes. *Proc. Natl Acad. Sci. USA* **113**, 310–315 (2016). 26712013 10.1073/pnas.1519401113 PMC4720338

[87] Schimmelpfennig, R. et al. The moderating role of culture in the generalizability of psychological phenomena. *Adv. Methods Pract. Psychol. Sci.* **7**, 25152459231225163 (2024).

[88] Zhou, H. & Fishbach, A. The pitfall of experimenting on the web: how unattended selective attrition leads to surprising (yet false) research conclusions. *J. Pers. Soc. Psychol.* **111**, 493–504 (2016). 27295328 10.1037/pspa0000056

[89] Chmielewski, M. & Kucker, S. C. An MTurk crisis? Shifts in data quality and the impact on study results. *Soc. Psychol. Pers. Sci.* **11**, 464–473 (2020).

[90] Aguinis, H., Villamor, I. & Ramani, R. S. MTurk research: review and recommendations. *J. Manag.* **47**, 823–837 (2021).

[91] Brodeur, A., Cook, N. & Heyes, A. *We Need to Talk About Mechanical Turk: What 22,989 Hypothesis Tests Tell Us About Publication Bias and P-Hacking in Online Experiments* Discussion Paper No. 15478 (IZA Institute of Labor Economics, 2022).

[92] Peer, E., Rothschild, D., Gordon, A., Evernden, Z. & Damer, E. Data quality of platforms and panels for online behavioral research. *Behav. Res. Methods* **54**, 1643–1662 (2022). 34590289 10.3758/s13428-021-01694-3 PMC8480459

[93] Webb, M. A. & Tangney, J. P. Too good to be true: bots and bad data from Mechanical Turk. *Perspect. Psychol. Sci*. 10.1177/17456916221120027 (2022). 10.1177/17456916221120027 36343213

[94] Douglas, B. D., Ewell, P. J. & Brauer, M. Data quality in online human-subjects research: comparisons between MTurk, Prolific, CloudResearch, Qualtrics, and SONA. *PLoS ONE* **18**, e0279720 (2023). 36917576 10.1371/journal.pone.0279720 PMC10013894

[95] Abelson, R. P. *Statistics as Principled Argument* (Psychology Press, 1995).

[96] Macdonald, R. R. Statistical inference and Aristotle’s Rhetoric. *Br. J. Math. Stat. Psychol.* **57**, 193–203 (2004). 15511303 10.1348/0007110042307186

[97] Scheel, A. M., Schijen, M. R. M. J. & Lakens, D. An excess of positive results: comparing the standard psychology literature with registered reports. *Adv. Methods Pract. Psychol. Sci.* **4**, 25152459211007467 (2021).

[98] Soderberg, C. K. et al. Initial evidence of research quality of registered reports compared with the standard publishing model. *Nat. Hum. Behav.* **5**, 990–997 (2021). 34168323 10.1038/s41562-021-01142-4

[99] Brodeur, A., Cook, N., Hartley, J. & Heyes, A. Do pre-registration and pre-analysis plans reduce p-hacking and publication bias? Evidence from 15,992 test statistics and suggestions for improvement. *JPE Micro.* **2**, 527–561 (2024).

[100] Yamada, Y. How to crack pre-registration: toward transparent and open science. *Front. Psychol*. **9**, 1831 (2018). 10.3389/fpsyg.2018.01831 PMC6168681 30319516

[101] Flis, I. The function of literature in psychological science. *Rev. Gen. Psychol.* **26**, 146–156 (2022).

[102] Rubin, M. Questionable metascience practices. *JOTE*10.36850/mr4 (2023).

[103] Wagenmakers, E.-J., Wetzels, R., Borsboom, D., van der Maas, H. L. J. & Kievit, R. A. An agenda for purely confirmatory research. *Perspect. Psychol. Sci.* **7**, 632–638 (2012). 26168122 10.1177/1745691612463078

[104] Nosek, B. A., Ebersole, C. R., DeHaven, A. C. & Mellor, D. T. The preregistration revolution. *Proc. Natl Acad. Sci. USA* **115**, 2600–2606 (2018). 29531091 10.1073/pnas.1708274114 PMC5856500

[105] Maxwell, S. E., Lau, M. Y. & Howard, G. S. Is psychology suffering from a replication crisis? What does ‘failure to replicate’ really mean? *Am. Psychol.* **70**, 487–498 (2015). 26348332 10.1037/a0039400

[106] Shrout, P. E. & Rodgers, J. L. Psychology, science, and knowledge construction: broadening perspectives from the replication crisis. *Annu. Rev. Psychol.* **69**, 487–510 (2018). 29300688 10.1146/annurev-psych-122216-011845

[107] Dreber, A. & Johannesson, M. A framework for evaluating reproducibility and replicability in economics. *Econ. Inq*. 10.1111/ecin.13244 (2024).

[108] Benjamin, D. J. et al. Redefine statistical significance. *Nat. Hum. Behav.* **2**, 6–10 (2018). 30980045 10.1038/s41562-017-0189-z

[109] Barnard, G. A. Significance tests for 2 × 2 tables. *Biometrika* **34**, 123–138 (1947). 20287826 10.1093/biomet/34.1-2.123

[110] Mehrotra, D. V., Chan, I. S. F. & Berger, R. L. A cautionary note on exact unconditional inference for a difference between two independent binomial proportions. *Biometrics* **59**, 441–450 (2003). 12926729 10.1111/1541-0420.00051

[111] Boschloo, R. D. Raised conditional level of significance for the 2 x 2-table when testing the equality of two probabilities. *Stat. Neerl.* **24**, 1–9 (1970).

[112] Simonsohn, U. Small telescopes: detectability and the evaluation of replication results. *Psychol. Sci.* **26**, 559–569 (2015). 25800521 10.1177/0956797614567341

[113] Ly, A., Verhagen, J. & Wagenmakers, E.-J. Harold Jeffreys’s default Bayes factor hypothesis tests: explanation, extension, and application in psychology. *J. Math. Psychol.* **72**, 19–32 (2016).

[114] Ly, A., Etz, A., Marsman, M. & Wagenmakers, E.-J. Replication Bayes factors from evidence updating. *Behav. Res. Methods* **51**, 2498–2508 (2019). 30105445 10.3758/s13428-018-1092-x PMC6877488

[115] Jeffreys, H. *The Theory of Probability* (Oxford Univ. Press, 1961).

[116] Patil, P., Peng, R. D. & Leek, J. T. What should researchers expect when they replicate studies? A statistical view of replicability in psychological science. *Perspect. Psychol. Sci.* **11**, 539–544 (2016). 27474140 10.1177/1745691616646366 PMC4968573

[117] Gelman, A. & Stern, H. The difference between ‘significant’ and ‘not significant’ is not itself statistically significant. *Am. Stat.* **60**, 328–331 (2006).

[118] Cumming, G. Replication and P intervals: P values predict the future only vaguely, but confidence intervals do much better. *Perspect. Psychol. Sci.* **3**, 286–300 (2008). 26158948 10.1111/j.1745-6924.2008.00079.x

[119] Muradchanian, J., Hoekstra, R., Kiers, H. & van Ravenzwaaij, D. How best to quantify replication success? A simulation study on the comparison of replication success metrics. *R. Soc. Open Sci.* **8**, 201697 (2021). 34017596 10.1098/rsos.201697 PMC8131945

[120] Altmejd, A. et al. Predicting the replicability of social science lab experiments. *PLoS ONE* **14**, e0225826 (2019). 31805105 10.1371/journal.pone.0225826 PMC6894796

[121] Yang, Y., Youyou, W. & Uzzi, B. Estimating the deep replicability of scientific findings using human and artificial intelligence. *Proc. Natl Acad. Sci. USA* **117**, 10762–10768 (2020). 32366645 10.1073/pnas.1909046117 PMC7245108

[122] Rajtmajer, S. et al. A synthetic prediction market for estimating confidence in published work. *Proc. AAAI Conf. Artif. Intell.* **36**, 13218–13220 (2022).

[123] Youyou, W., Yang, Y. & Uzzi, B. A discipline-wide investigation of the replicability of psychology papers over the past two decades. *Proc. Natl Acad. Sci. USA* **120**, e2208863120 (2023). 36716367 10.1073/pnas.2208863120 PMC9963456

[124] Agley, J., Xiao, Y., Nolan, R. & Golzarri-Arroyo, L. Quality control questions on Amazon’s Mechanical Turk (MTurk): a randomized trial of impact on the USAUDIT, PHQ-9, and GAD-7. *Behav. Res. Methods* **54**, 885–897 (2022). 34357539 10.3758/s13428-021-01665-8 PMC8344397

[125] Veselovsky, V., Ribeiro, M. H. & West, R. Artificial artificial artificial intelligence: crowd workers widely use large language models for text production tasks. Preprint at https://arxiv.org/abs/2306.07899 (2023).

[126] Olsson-Collentine, A., Wicherts, J. M. & van Assen, M. A. L. M. Heterogeneity in direct replications in psychology and its association with effect size. *Psychol. Bull.* **146**, 922–940 (2020). 32700942 10.1037/bul0000294

[127] Linden, A. H. & Hönekopp, J. Heterogeneity of research results: a new perspective from which to assess and promote progress in psychological science. *Perspect. Psychol. Sci.* **16**, 358–376 (2021). 33400613 10.1177/1745691620964193 PMC7961629

[128] Holzmeister, F. et al. Heterogeneity in effect size estimates. *Proc. Natl Acad. Sci. USA* **121**, e2403490121 (2024). 39078672 10.1073/pnas.2403490121 PMC11317577

[129] Moshontz, H. et al. The Psychological Science Accelerator: advancing psychology through a distributed collaborative network. *Adv. Methods Pract. Psychol. Sci.* **1**, 501–515 (2018). 31886452 10.1177/2515245918797607 PMC6934079

[130] Epstein, R. & Robertson, R. E. The search engine manipulation effect (SEME) and its possible impact on the outcomes of elections. *Proc. Natl Acad. Sci. USA* **112**, E4512–E4521 (2015). 26243876 10.1073/pnas.1419828112 PMC4547273

[131] Gallo, E. & Yan, C. The effects of reputational and social knowledge on cooperation. *Proc. Natl Acad. Sci. USA* **112**, 3647–3652 (2015). 25775544 10.1073/pnas.1415883112 PMC4378402

[132] Li, V., Michael, E., Balaguer, J., Herce Castañón, S. & Summerfield, C. Gain control explains the effect of distraction in human perceptual, cognitive, and economic decision making. *Proc. Natl Acad. Sci. USA* **115**, E8825–E8834 (2018). 30166448 10.1073/pnas.1805224115 PMC6156680

[133] Hanson, R. Logarithmic market scoring rules for modular combinatorial information aggregation. *J. Predict. Mark.* **1**, 3–15 (2007).

