# Supplementary material for: Examining the replicability of online experiments selected by a decision market
Source: Nat Hum Behav. 2024 Nov 19;9(2):316–30. doi: 10.1038/s41562-024-02062-9 (PMC11860227; doi:10.1038/s41562-024-02062-9)
Supplement: Supplementary file 1 — Supplementary Notes, references, Fig. 1 and Tables 1–8. [file 41562_2024_2062_MOESM1_ESM.pdf]

nature human behaviour

Supplementary information https://doi.org/10.1038/s41562-024-02062-9

Examining the replicability of online experiments selected by a decision market

In the format provided by the authors and unedited

Supplementary Notes **1**
Replication Sample Sizes 1
Conversion of Effect Sizes to Cohen's d 2
Preregistered Hypothesis Tests and Exploratory Analyses 3
Protocol Deviations and Further Information on the 26 Replications 5
Supplementary References **12**
Supplementary Figures **15**
Supplementary Tables **16**

Supplementary Notes

Below we provide further details on the replication sample sizes, the conversion of effect sizes to Cohen's *d*, the preregistered hypothesis tests and exploratory analyses, and deviations from the 26 preregistered analysis plans for the 26 replications.

Replication Sample Sizes

The replications of the 26 studies, selected from the sample of 41 studies[1–41] published in the *Proceedings of the National Academy of Sciences* based on the final market prices in the decision market, were carried out with high statistical power. Replication sample sizes were based on having 90% power to detect ⅔ of the effect size reported in the original study (with the effect size converted to Cohen's *d* to have a common standardized effect size measure across the original studies and the replication studies). The criteria for replication were an effect in the same direction as the original study and $p < 0.05$ (in a two-sided test). In cases where this power estimation led to a sample size smaller than the original one, we used the same sample size as in the original study. On average, the replication sample size was 3.5 times as large as the original study sample size (the average sample size in the 26 original studies selected for publication was 292, and the average sample size in the replications was 1,019).

We used the following formula to estimate the "replication sample size factor (*f*)," i.e., the factor by which the original sample size must be multiplied to have 90% power to detect ⅔ of the original effect size in a two-sided test at the 5% significance level.

$$f = \left(\frac{\phi^{-1}(0.975) + \phi^{-1}(0.90)}{\frac{2}{3} \cdot z} \right)^2 = \left(\frac{3.2415\ldots}{\frac{2}{3} \cdot z} \right)^2$$

where ϕ^{-1} denotes the inverse cumulative distribution function of a standard normal random variable, i.e., $\phi^{-1}(0.975)$ and $\phi^{-1}(0.90)$ refers to the critical value of a two-tailed 5% threshold and a one-tailed 10% threshold, respectively. The first critical value is the critical value at the 5% significance level; the second critical value is the addition needed to have 90% power (if the true effect size divided by the standard error is 3.2415, there is a 10% probability that the observed effect size will yield a *z*-value below 1.96 and a 90% probability that it will yield a *z*-value above 1.96). The value $\phi^{-1}(0.975) + \phi^{-1}(0.90) = 3.2415$ equals the factor one would multiply the standard errors with to get the minimum effect size detectable with 90% power at the 5% significance level.

In the above formula, *z* is the *z*-value of the original study for the hypothesis test being replicated. This formula gives the factor to multiply the original sample size to get the replication sample size (e.g., if $f = 3$ and the original study included 100 observations, the replication sample size will be 300). For a *t*-test, we replace *z* with *t* in the formula above (and $F(1, df)$ *F*-tests and $\chi^2(1)$ tests are converted to *t* and *z* values as for the conversion to Cohen's *d*; see below). The replication sample formula was based on the relationship between *n* and the

standard error (*se*) in a standard *z*-test and *t*-test (and this relationship is the same in an independent-samples test and a paired test). When the "replication sample factor" (*f*) in the formula above was below 1, we set the replication sample size to the same as the original one (as ⅔ of the original effect size implies a smaller replication sample size); this ensured that no replication study had a smaller sample size than in the original study. In these cases, the power to detect ⅔ of the original effect size in the replication study exceeded 90%, which happened for seven studies[5,11,17,28,30,32,40].

Conversion of Effect Sizes to Cohen's *d*

We converted the effect sizes of all the original studies and all the replication studies to Cohen's *d* to have a standardized effect size measure. The effect size estimates for the original studies are always assigned a positive sign; the effect sizes in the replication studies are assigned a positive sign if a replication effect points in the same direction as in the original study and a negative sign if the effect points in the opposite direction. The estimations of Cohen's *d* were based on the formula used by Szucs & Ioannidis[42] (Supplementary Materials, pp. 2–3) to convert test statistics obtained from independent-samples *t*-tests and paired *t*-tests to Cohen's *d* units. They used the following two formulas:

Unpaired *t*-test: $d = \frac{2t}{\sqrt{n}}$

Paired *t*-test: $d = \frac{t}{\sqrt{n}}$

In the two above formulas, *n* is the sample size in the study (the number of individuals for studies that use tests based on the individual data and the number of groups for studies that use tests based on d ata aggregated on the group level). Note that the paired *t*-test formula assumes a 0.5 correlation in observations within pairs (and the formula for the independent-samples *t*-test assumes equal sample sizes in group 1 and group 2).

We used the unpaired *t*-test formula for studies using between-subjects tests and the paired *t*-test formula for studies using within-subject tests. *F(1, df)* test statistics were converted to *t*-values by taking the square root. Studies based on *z*-test statistics were converted to Cohen's *d* using the same formulas as above but replacing *t* with *z*. $\chi^2(1)$ statistics were converted to *z*-statistics by taking the square root.

For interactions between two between-subjects factors, the above unpaired *t*-test formula will underestimate effect sizes. Therefore, for those studies, we used the following formula to convert effect size estimates to Cohen's *d* units:

Interactions of two between factors: $d = \frac{4t}{\sqrt{n}}$

The basis of the above formula is that an interaction test of two between-subjects factors will increase the standard error by a factor of about two as compared to an estimate of the main

effects. The following five studies estimated an interaction between two between-subjects factors: Clarkson et al.[10], Côté et al.[12], Handley et al.[18], Baldwin & Lammers[3], and Hoffman et al.[19].

There were also two studies that interacted one between-subjects variable with one within-subject variable. These two studies were converted to Cohen's *d* using the unpaired *t*-test formula (and we coded these studies as "between subjects tests" and "interaction tests" in the study). These two studies are Bear et al.[4] and Morris et al.[32].

We also have one study that interacted two within-subject factors. This study was converted to Cohen's *d* using the paired *t*-test formula. This study is Cooney et al.[11].

Standard errors of Cohen's d and confidence intervals. The *t* and *z* test statistic shows the ratio between the effect size and the standard error. We derived the standard error of Cohen's *d* by preserving this ratio (e.g., if a study with a *t*-value of 2 was converted to a Cohen's *d* of 1, the standard error of Cohen's *d* is 0.5). The 95% confidence intervals of Cohen's *d* for studies using *t*-tests (or *F*-tests converted to a *t*-test statistic) were estimated as $d \pm se \cdot t^{-1}(0.025)$; where $t^{-1}(0.025)$ denotes the critical value of the inverse *t*-distribution (for the *df* of the *t*-test) at 2.5%, i.e., the 5% threshold in a two-sided test. The 95% confidence intervals of Cohen's *d* for studies using *z*-test statistics (or χ^2 tests converted to a *z*-test statistic) were estimated as $d \pm se \cdot \phi^{-1}(0.025)$, where $\phi^{-1}(0.025)$ denotes the critical value of the inverse standard normal distribution at 2.5%, i.e., the 5% threshold in a two-sided test.

Preregistered Hypothesis Tests and Exploratory Analyses

The hypothesis tests were divided into primary and secondary hypothesis tests. All hypothesis tests were based on two-tailed *p*-values. We interpret a *p*-value below 0.5% as "statistically significant evidence" and a *p*-value below 5% as "suggestive evidence" following the recommendation of Benjamin et al.[43].

Primary hypothesis 1: There is a positive correlation between the decision market prices and the replication outcomes for the 26 replicated studies.

This was tested using a point-biserial correlation between the final decision market prices and the replication outcomes based on the statistical significance criterion. We think of this as a test of "proof of concept" of decision markets. For markets to be used as a tool to select studies to be replicated, they need to be able to predict replication outcomes to some extent, implying a positive correlation between market prices and replication outcomes. Such a positive correlation has been found in previous large-scale replication projects, but it is not obvious that those results carry over to decision markets. This test result is reported in the main text.

Primary hypothesis 2: The standardized effect size (measured in terms of Cohen's *d*) is lower in the 26 replication studies than in the 26 original studies.

This was tested using a Wilcoxon signed-ranks test of the replication effect sizes versus the original effect sizes for the 26 replication studies. Previous large-scale replication studies have found that the replication effect sizes are, on average, about 50% of the original studies, and we expected to observe similar replication effect sizes in this study. This test result is reported in the main text.

Secondary hypothesis 1: The replication rate is lower among the 12 studies with the lowest decision market prices than for the 12 studies with the highest decision market prices.

This was tested using Fisher's exact test comparing the replication rate using the statistical significance criterion between the 12 studies with the lowest decision market prices and the 12 studies with the highest decision market prices. This test is related to primary hypothesis 1 and is an alternative "proof of concept" test of decision markets, but as it has somewhat lower power, we included it as a secondary hypothesis test. This test result is reported in the main text.

Secondary hypothesis 2: There is a positive correlation between the average survey belief of replication and the replication outcomes for the 26 replicated studies.

This was tested using a point-biserial correlation between the average survey belief about replication and the replication outcomes based on the statistical significance criterion. This tests if the survey responses can predict replications and corresponds to the primary hypothesis 1 test, but using survey data instead of decision market prices. The average survey-predicted probability of replication for each of the 26 replication studies was estimated for those survey respondents who did at least one trade on the decision market. This test result is reported in the main text.

Secondary hypothesis 3: There is a positive correlation between the average survey belief of replication and the decision market prices for the 26 replicated studies.

This was tested using a Pearson correlation between the average survey belief about replication and the final market prices. This tests if the predictions of the decision market and the survey are correlated. As above, the average survey-predicted probability of replication for each of the 26 replication studies was estimated for those survey respondents who made at least one trade on the decision market. This test result is reported in the main text.

Secondary hypothesis 4a: The average absolute prediction error is lower for the decision market than for the survey for the 26 replicated studies.

This was tested using a Wilcoxon signed-ranks test defining the absolute prediction error as the absolute difference between the prediction and the replication outcome based on the statistical significance criterion. This tests if the decision market outperforms the survey in predicting replication outcomes. As above, the average survey-predicted probability of replication for each of the 26 replication studies was estimated for those survey respondents who made at least one trade on the decision market. This test result is reported in the main text.

Secondary hypothesis 4b: The average squared prediction error (Brier score) is lower for the decision market than for the survey for the 26 replicated studies.

This was tested using a Wilcoxon signed-ranks test of the squared prediction error (the Brier score). This is a test of the same hypothesis as in secondary hypothesis 4a, but using the squared prediction error (Brier score) instead of the absolute prediction error to measure prediction performance. As above, the average survey-predicted probability of replication for each of the 26 replication studies was estimated for those survey respondents who made at least one trade on the decision market. This test result is reported in the main text.

Preregistered Exploratory analyses: In the exploratory analyses, we tested if the average belief (measured on a scale from −3 to 3) about whether the pandemic has affected the probability of replication differed from zero in a one-sample *t*-test; this test was carried out separately for each of the 26 replication studies. We also tested if the average across the 26 replication studies differed from zero in a one-sample *t*-test (i.e., we first constructed the average answer for the 26 questions for each survey respondent so that we have one observation per respondent and then tested if the average of this variable differed from zero). These test results are reported in the main text and Supplementary Table 5.

We additionally tested if the average belief about whether the pandemic has affected the probability of replication was significantly correlated with the replication outcomes based on the statistical significance criterion using a point-biserial correlation; and if it was significantly correlated with the final decision market prices and the average survey belief of replication based on a Pearson correlation. The number of observations for estimating these correlations was 26. In all these exploratory analyses, only data from those survey respondents who did at least one trade on the decision market were included. These test results are reported in the main text.

Protocol Deviations and Further Information on the 26 Replications

Prior to starting the survey data collection (that preceded the decision market and replications), we preregistered an analysis plan (a replication report) for each of the 41 potential replications at OSF after obtaining feedback from the original authors. After the replications, the 26 replication reports of the implemented replications were updated with the results of the replications and also posted at OSF. Furthermore, we provided all original authors the opportunity to comment on the replications (without a particular due date) and make the comments available as we receive them alongside the replication reports. The preregistered replication reports, the post-replication reports, and the original authors commentaries (if available) are available at https://osf.io/sejyp.

Below we mention any deviations from the preregistered designs and analyses and further information on the implementation of the replication experiments for the 26 individual

replications (in case there were any issues with the implementation). Deviations from the protocol are also detailed in the individual replication reports for each replication posted at OSF:

- **Atir and Ferguson**[2]**:** We preregistered to perform the same analysis as in the original article, i.e., a test of fixed effects in a mixed-effects model. While we followed the pre-registered analysis exactly, the replication result is based on a *z*-test (rather than an *F*-test as reported in the original article) due to differences in the optimization routines implemented in different software applications (the replication result has been estimated in Stata, the original result in SPSS).
- **Cheon and Hong**[9]**:** The original authors discovered an error in the reference to the ANOVA in the "Hypothesis to replicate and bet on" section of the replication report when giving feedback on the replication results. The pre-replication version of the report erroneously reported the ANOVA result from Study 3 (instead of Study 2) of the original article. But the focal test for the replication (a *t*-test), provided by the authors since it is not reported in their article, was correctly reported in the "Hypothesis to replicate and bet on" section. Thus, the reporting error does neither affect the replication design nor the data analysis.
- **Côté et al.**[12]**:** We erroneously preregistered that income would enter the regression analysis in terms of a dichotomized variable. For the analysis of the replication data, we follow the original article and use participants' continuous income reports (mean-centered) instead.
- **Gheorghiu et al.**[15]**:** Fifteen participants were excluded from the analysis due to technical issues (e.g., problems with loading pages). We preregistered that the focal test is based on the *t*-test of the coefficient of interest in a mixed-effects regression. While we followed the pre-registered analysis exactly, the replication result is based on a *z*-test due to differences in the optimization routines implemented in different software applications (the replication result has been estimated in Stata, the original result in R).
- **Guilbeault et al.**[16]**:** The replication test is carried out on the network level, where each network results in one observation. We planned to collect 56 networks (28 network observations per treatment). Furthermore, we planned to include 40 individual participants per network so that the total number of participants would be 56 x 40 = 2,240. However, we did not manage to have 40 participants in all networks; eventually, we only had 2,001 participants across 56 networks (i.e., we reached the planned sample size in terms of the number of networks that is the unit of observation in the analysis, but the number of participants per network was lower than planned). We conducted 28 sessions with two networks each, but it was challenging to recruit and perpetuate exactly 40 participants per network, so we decided to start the experiment whenever we had what we deemed was a sufficient number of participants. The median network size was 37, with the smallest network having 30 participants. Not all

participants completed the whole three rounds of the experiment. The main variable of interest is the percentage of participants who update their opinions between rounds 1 and 3; when we count the number of participants who entered their guesses in both rounds 1 and 3, we have a sample of 1,764 participants. We note that the original study—although not explicitly mentioned in the paper—did not have exactly 40 participants per network either. The original data comprises 890 participants instead of 24 networks x 40 = 960 participants; restricting our attention to those who entered their guesses in round 1 and round 3, the number goes down to 858.

- **Hofstetter et al.**[20]**:** Since the implementation in Qualtrics and PHP proved to be somewhat cumbersome and unreliable, the experiment was closely reprogrammed in oTree[44] based on the materials provided by the original authors. Before starting the replication, the final software was shared with the original authors, who approved it.
- **John et al.**[24]**:** The final sample size of 1,224 considerably exceeds the targeted sample of 776. As per the pre-registration, we planned to collect data on a third condition ("revealer") for exploratory purposes, which resulted in an overall targeted number of observations of 1,164 (776 of which pertain to the two focal treatment conditions used in the replication test). Accidentally, we only collected data for the two focal treatment conditions, which resulted in an oversampling of around 50%. Consequently, the statistical power to detect ⅔ of the original effect size is 98.3% rather than the targeted 90.0%. As a consequence of the failure to collect data on all three conditions, we deviate from the preregistered analysis plan in so far as we cannot report the exploratory results on the comparisons between the "revealer" condition and the "hider" and "inadvertent nondiscloser" condition, respectively.
- **Jordan et al.**[25]**:** Due to a misunderstanding, we initially collected player A data only (i.e., only data on which the focal test is based), which entailed deception. When we realized the misunderstanding, we decided not to conduct any analyses based on these data but to collect new data on both players A and B (hence, avoiding deception) as in the original article and as planned as per our preregistered protocol. This decision was made without inspecting the data from the first batch and was approved by the original authors. Participants from the first batch were not allowed to participate in the second and final batch (and the data collection was carried out using a separate MTurk account). All results in the main text are based on the second data collection carried out as preregistered (i.e., not involving deception).

  Once we informed the original authors about the replication results (based on the second data collection), they requested to get access to the data from the first batch data collection, which we shared with them via e-mail (still without inspecting these data or running any analyses). The original authors analyzed the first-batch data and found that the original result replicated according to the statistical significance indicator: $z = 2.222$, $p = 0.026$, $n = 3{,}628$; $d = 0.074$, 95% CI [0.009, 0.139]; relative replication

effect size = 32.1% (as compared to the replication results of the second-batch data collection included in the main text: $z = 1.062$, $p = 0.288$, $n = 1,826$; $d = 0.050$, 95% CI [−0.042, 0.141]; relative replication effect size = 21.6%).

Several things should be noted about these results pertaining to the first-batch data collection. First, the results are based on about twice the planned replication sample size ($n = 3,628$ instead of the planned sample of $n = 1,791$), resulting from the mistake of collecting only player A data instead of both player A and B data. Note that the doubled sample size implies a substantial increase in statistical power: particularly, the sample of $n = 3,628$ implies 99.6% power to detect an effect of $d = 0.153$ (= ⅔ of the original effect size); put differently, holding power constant at the designated level of 90%, the minimum detectable effect size is substantially reduced to $d = 0.108$ (< 50% of the original effect size). If the planned replication sample size had been collected, the replication would most likely not have been successfully replicated according to the statistical significance indicator ($p < 0.05$ and an effect in the original direction). To illustrate that the significant result in the first-batch data collection is an “artifact” of the lower minimum detectable effect size due to the doubled sample size, we randomly drew 1,791 observations from the $n = 3,628$ dataset 10,000 times: only in 28.1% of these draws, the focal hypothesis test resulted in $p < 0.05$ and an effect in the same direction as in the original paper (effect sizes [in Cohen’s *d* units] across the 10,000 iterations range from −0.062 to 0.189, with a mean of 0.073). Second, conducting the replication test also based on the first-batch data implies that two independent tests of replication would be conducted, doubling the false positive risk of the test. Adjusting for family-wise errors (using a Bonferroni correction), the result does not replicate according to the statistical significance indicator ($p = 0.026 > \alpha' = 0.025$) even if all the $n = 3,628$ observations are included in the analysis. Third, even if we had included the first data collection with all $n = 3,628$ observations instead of the second data collection, our overall results and conclusions would barely change, except for the evidence in support of the first primary hypothesis would change from “suggestive” to “significant” (see below). The relative effect size of the Jordan et al. replication would have increased from 21.6% to 32.1%, and the replication rate (based on the statistical significance indicator) would have increased from 53.8% to 57.7%. The first average effect size measure would increase from 45.0% to 45.1% (with the difference between original and replication effect size, i.e., our test of the second primary hypothesis, still being statistically significant; Wilcoxon signed-rank test: $z = 4.178$, $p < 0.001$, $n = 26$) and the second relative effect size measure would increase from 41.1% to 41.5%. For the first primary hypothesis, the correlation between decision market prices and replication outcomes with the statistical significance indicator would change from $r = 0.505$ (95% CI [0.146, 0.712]; $t(24) = 2.867$, $p = 0.008$; $n = 26$) to $r = 0.549$ (95% CI [0.206, 0.738]; $t(24) = 3.221$ $p = 0.004$; $n = 26$).

- **Kouchaki & Gino[29]:** In addition to the preregistered exclusion criteria, we chose to exclude participants who failed to self-report the number of words they found in the

boggle task (even if they entered one or more correct words in the subsequent stage of the experiment) since these participants can neither be classified as “overreporters” nor as “underreported.” The original article is not specific about how these cases were dealt with, and we failed to foresee that this would likely happen during data collection (i.e., this choice was not pre-registered). Yet, we deem this choice suitable in the context of the hypothesis and consider it more like a necessary ex-post decision rather than a deviation from the pre-analysis plan. Furthermore, the pre-registration failed to explicate that only *correctly* identified words are counted; words longer/shorter than four characters and words that could not be constructed from the letter matrix were ignored.

- **Morris et al.**[32]: After we had conducted the replication of Morris et al. and sent the replication report to the original authors for feedback, the original authors alerted us that they had discovered an error in the analysis code of the original study. In the original article, the authors state that the hypothesis is tested through the interaction effect between role and round in a mixed-effects model controlling for game order using a likelihood ratio test ($\chi^2(1) = 24.3$, $p < 0.001$, $n = 100$, $d = 0.986$). Controlling for game order was achieved by adding the game order variable as a covariate to the model and interacting it with all other covariates in the model, which was not explicated in the original paper. Since the pre-registration for the replication of the study was based on the information available in the original article, the focal test was thus described as a test of the interaction effect of role and round in a mixed-effects model (evaluated through a likelihood ratio test). This information was also provided to forecasters in the prediction survey and the decision market.

  By mistake, the original authors dummy-coded the game order variable instead of effect-coding it, implying that the estimate of the interaction term of role and round pertains to one specific game order (the game order coded as 0) rather than to the average of both game orders. We used the analysis code provided by the original authors and, therefore, initially implemented the exact same test as the original authors. Based on this initial analysis, the study did not replicate: $\chi^2(1) = 0.84$, $p = 0.359$, $d = 0.162$. According to personal correspondence with the original authors, they have already submitted a corrigendum to PNAS to correct the erroneous test result in the original study (so that it is based on effect-coding the game order variable). As we interpret this as an unintentional coding error in the original study, we use the corrected code (based on effect-coding the game order variable instead of dummy-coding the variable) as our replication test (as the corrected test is in line with how the test is described in our preregistered replication report and described to forecasters in the survey and the decisions market).

  Based on the corrected analysis code, the study replicates according to the statistical significance indicator ($\chi^2(1) = 5.495$, $p = 0.0191$, $n = 128$, $d = 0.414$). An implication of the coding error in the original study is that the original result reported to decision market participants and included in the replication report is incorrect; the original result based on

the corrected code (with effect coding of the game order variable) is: $\chi^2(1) = 12.979$, $p < 0.001$, $d = 0.721$. As the power calculation and replication sample size calculation was based on the incorrect original result, the actual replication power based on the corrected analysis code is also less than the planned 90% power to detect ⅔ of the original effect size. The replication power (for the replication sample of $n = 128$) based on the corrected original result is 77.6% to detect ⅔ of the original effect size. If we had instead used the exact same erroneous replication test as used in the original study (i.e., dummy-coding the game order variable), the relative effect size of the Morris et al. replication would decrease from 57.5% to 16.4%, and the replication rate (based on the statistical significance indicator) would decrease from 53.8% to 50.0%. The first average effect size measure would decrease from 45.0% to 42.5% (with the difference between original and replication effect size, i.e., our test of the second primary hypothesis, being unaffected; Wilcoxon signed-rank test: $z = 4.203$, $p < 0.001$, $n = 26$) and the second relative effect size measure would decrease from 41.1% to 39.5%. For the first primary hypothesis, the correlation between decision market prices and replication outcomes with the statistical significance indicator would change from $r = 0.505$ (95% CI [0.146, 0.712]; $t(24) = 2.867$, $p = 0.008$; $n = 26$) to $r = 0.425$ (95% CI [0.043, 0.663]; $t(24) = 2.297$, $p = 0.031$; $n = 26$).

- **Reeck et al.[37]:** We collected data for this replication in two batches. In the first batch, we collected data from 1412 participants. Due to errors in the preprocessing code, initially, fewer participants were excluded (and subsequently analyzed) than would have been correct based on the exclusion criteria. To debug the code, another team member implemented the preprocessing pipeline independently and resolved discrepancies in the results through discussion with the replication team. When the errors were corrected, 864 valid responses were left after exclusion, which is less than the target sample size. We then collected data from an additional 288 participants, from which 180 valid responses remained after exclusion.
  The analysis code provided by the authors only covered the main analysis but not the data preprocessing. The code itself was written by the replication team after the data was collected and should not be considered formally preregistered. We used the same preprocessing procedure as described in the original manuscript. In addition to the preregistered exclusion criteria, we excluded *(i)* participants who quit the session during the introduction round (and, thus, had no valid data to analyze; $n = 91$); *(ii)* participants who ran the experiment on a mobile device or a tablet ($n = 2$); and *(iii)* participants who quit the task before it was completed (and, thus, had no valid completion code; $n = 198$). Since the experiment involves tracking mouse movements, excluding participants running the study on a smartphone or tablet was a requirement. The original study does not explicate how these cases were dealt with. Note that the advertisement explained that participants must use a computer, not any mobile device. Due to a mistake, the attention check that all participants in our replication studies had to pass to proceed to

the replication study (see Methods for details) was not implemented for this replication study (the Captcha and the IP quality checks were also implemented for this replication, though).

The original authors disagreed about the exact steps in preprocessing the replication data and sent us the code for their preferred preprocessing of the replication data (and the replication results based on their preferred preprocessing). The original authors' preferred preprocessing resulted in more exclusions of observations and a sample size of $n = 857$; but the replication result based on their preferred preprocessing is similar to our reported replication results (the result based on the preprocessing preferred by the original authors (based on the original authors' estimation) is: $z = 1.111$, $p = 0.267$, $d = 0.076$, 95% CI [−0.058, 0.210]; our reported replication result is: $z = 1.084$, $p = 0.278$, $d = 0.067$, 95% CI [−0.054, 0.188]). It should be noted that the data preprocessing in this study was complicated due to ambiguity about the exact preprocessing steps in the original paper (as we did not have access to the raw data or the preprocessing code used in the original study).

Supplementary References

1. Ames, D. L. & Fiske, S. T. Perceived intent motivates people to magnify observed harms. *Proc. Natl. Acad. Sci.* **112**, 3599–3605 (2015).
2. Atir, S. & Ferguson, M. J. How gender determines the way we speak about professionals. *Proc. Natl. Acad. Sci.* **115**, 7278–7283 (2018).
3. Baldwin, M. & Lammers, J. Past-focused environmental comparisons promote proenvironmental outcomes for conservatives. *Proc. Natl. Acad. Sci.* **113**, 14953–14957 (2016).
4. Bear, A., Fortgang, R. G., Bronstein, M. V. & Cannon, T. D. Mistiming of thought and perception predicts delusionality. *Proc. Natl. Acad. Sci.* **114**, 10791–10796 (2017).
5. Boswell, R. G., Sun, W., Suzuki, S. & Kober, H. Training in cognitive strategies reduces eating and improves food choice. *Proc. Natl. Acad. Sci.* **115**, E11238–E11247 (2018).
6. Caruso, E. M., Burns, Z. C. & Converse, B. A. Slow motion increases perceived intent. *Proc. Natl. Acad. Sci.* **113**, 9250–9255 (2016).
7. Casella, A., Kartik, N., Sanchez, L. & Turban, S. Communication in context: Interpreting promises in an experiment on competition and trust. *Proc. Natl. Acad. Sci.* **115**, 933–938 (2018).
8. Chao, M. Demotivating incentives and motivation crowding out in charitable giving. *Proc. Natl. Acad. Sci.* **114**, 7301–7306 (2017).
9. Cheon, B. K. & Hong, Y.-Y. Mere experience of low subjective socioeconomic status stimulates appetite and food intake. *Proc. Natl. Acad. Sci.* **114**, 72–77 (2017).
10. Clarkson, J. J. *et al.* The self-control consequences of political ideology. *Proc. Natl. Acad. Sci.* **112**, 8250–8253 (2015).
11. Cooney, G., Gilbert, D. T. & Wilson, T. D. When fairness matters less than we expect. *Proc. Natl. Acad. Sci.* **113**, 11168–11171 (2016).
12. Côté, S., House, J. & Willer, R. High economic inequality leads higher-income individuals to be less generous. *Proc. Natl. Acad. Sci.* **112**, 15838–15843 (2015).
13. Flesch, T., Balaguer, J., Dekker, R., Nili, H. & Summerfield, C. Comparing continual task learning in minds and machines. *Proc. Natl. Acad. Sci.* **115**, E10313–E10322 (2018).
14. Genschow, O., Rigoni, D. & Brass, M. Belief in free will affects causal attributions when judging others' behavior. *Proc. Natl. Acad. Sci.* **114**, 10071–10076 (2017).
15. Gheorghiu, A. I., Callan, M. J. & Skylark, W. J. Facial appearance affects science communication. *Proc. Natl. Acad. Sci.* **114**, 5970–5975 (2017).
16. Guilbeault, D., Becker, J. & Centola, D. Social learning and partisan bias in the interpretation of climate trends. *Proc. Natl. Acad. Sci.* **115**, 9714–9719 (2018).
17. Halevy, N. & Halali, E. Selfish third parties act as peacemakers by transforming conflicts and promoting cooperation. *Proc. Natl. Acad. Sci.* **112**, 6937–6942 (2015).
18. Handley, I. M., Brown, E. R., Moss-Racusin, C. A. & Smith, J. L. Quality of evidence

revealing subtle gender biases in science is in the eye of the beholder. *Proc. Natl. Acad. Sci.* **112**, 13201–13206 (2015).

19. Hoffman, K. M., Trawalter, S., Axt, J. R. & Oliver, M. N. Racial bias in pain assessment and treatment recommendations, and false beliefs about biological differences between blacks and whites. *Proc. Natl. Acad. Sci.* **113**, 4296–4301 (2016).
20. Hofstetter, R., Rüppell, R. & John, L. K. Temporary sharing prompts unrestrained disclosures that leave lasting negative impressions. *Proc. Natl. Acad. Sci.* **114**, 11902–11907 (2017).
21. Horne, Z., Powell, D., Hummel, J. E. & Holyoak, K. J. Countering antivaccination attitudes. *Proc. Natl. Acad. Sci.* **112**, 10321–10324 (2015).
22. Isley, S. C., Stern, P. C., Carmichael, S. P., Joseph, K. M. & Arent, D. J. Online purchasing creates opportunities to lower the life cycle carbon footprints of consumer products. *Proc. Natl. Acad. Sci.* **113**, 9780–9785 (2016).
23. Jachimowicz, J. M., Chafik, S., Munrat, S., Prabhu, J. C. & Weber, E. U. Community trust reduces myopic decisions of low-income individuals. *Proc. Natl. Acad. Sci.* **114**, 5401–5406 (2017).
24. John, L. K., Barasz, K. & Norton, M. I. Hiding personal information reveals the worst. *Proc. Natl. Acad. Sci.* **113**, 954–959 (2016).
25. Jordan, J. J., Hoffman, M., Nowak, M. A. & Rand, D. G. Uncalculating cooperation is used to signal trustworthiness. *Proc. Natl. Acad. Sci.* **113**, 8658–8663 (2016).
26. Jun, Y., Meng, R. & Johar, G. V. Perceived social presence reduces fact-checking. *Proc. Natl. Acad. Sci.* **114**, 5976–5981 (2017).
27. KC, R. P., Kunter, M. & Mak, V. The influence of a competition on noncompetitors. *Proc. Natl. Acad. Sci.* **115**, 2716–2721 (2018).
28. Klein, N. & O'Brien, E. People use less information than they think to make up their minds. *Proc. Natl. Acad. Sci.* **115**, 13222–13227 (2018).
29. Kouchaki, M. & Gino, F. Memories of unethical actions become obfuscated over time. *Proc. Natl. Acad. Sci.* **113**, 6166–6171 (2016).
30. Kraus, M. W., Rucker, J. M. & Richeson, J. A. Americans misperceive racial economic equality. *Proc. Natl. Acad. Sci.* **114**, 10324–10331 (2017).
31. McCall, L., Burk, D., Laperrière, M. & Richeson, J. A. Exposure to rising inequality shapes Americans' opportunity beliefs and policy support. *Proc. Natl. Acad. Sci.* **114**, 9593–9598 (2017).
32. Morris, A., MacGlashan, J., Littman, M. L. & Cushman, F. Evolution of flexibility and rigidity in retaliatory punishment. *Proc. Natl. Acad. Sci.* **114**, 10396–10401 (2017).
33. Mummolo, J. Militarization fails to enhance police safety or reduce crime but may harm police reputation. *Proc. Natl. Acad. Sci.* **115**, 9181–9186 (2018).
34. Payne, B. K., Brown-Iannuzzi, J. L. & Hannay, J. W. Economic inequality increases risk taking. *Proc. Natl. Acad. Sci.* **114**, 4643–4648 (2017).

35. Phillips, J. & Cushman, F. Morality constrains the default representation of what is possible. *Proc. Natl. Acad. Sci.* **114**, 4649–4654 (2017).

36. Rai, T. S., Valdesolo, P. & Graham, J. Dehumanization increases instrumental violence, but not moral violence. *Proc. Natl. Acad. Sci.* **114**, 8511–8516 (2017).

37. Reeck, C., Wall, D. & Johnson, E. J. Search predicts and changes patience in intertemporal choice. *Proc. Natl. Acad. Sci.* **114**, 11890–11895 (2017).

38. Schilke, O., Reimann, M. & Cook, K. S. Power decreases trust in social exchange. *Proc. Natl. Acad. Sci.* **112**, 12950–12955 (2015).

39. Stern, C., West, T. V. & Rule, N. O. Conservatives negatively evaluate counterstereotypical people to maintain a sense of certainty. *Proc. Natl. Acad. Sci.* **112**, 15337–15342 (2015).

40. Vacharkulksemsuk, T. *et al.* Dominant, open nonverbal displays are attractive at zero-acquaintance. *Proc. Natl. Acad. Sci.* **113**, 4009–4014 (2016).

41. Williams, K. E. G., Sng, O. & Neuberg, S. L. Ecology-driven stereotypes override race stereotypes. *Proc. Natl. Acad. Sci.* **113**, 310–315 (2016).

42. Szucs, D. & Ioannidis, J. P. A. Empirical assessment of published effect sizes and power in the recent cognitive neuroscience and psychology literature. *PLoS Biol.* **15**, e2000797 (2017).

43. Benjamin, D. J. *et al.* Redefine statistical significance. *Nat. Hum. Behav.* **2**, 6–10 (2018).

44. Chen, D. L., Schonger, M. & Wickens, C. oTree—An open-source platform for laboratory, online, and field experiments. *J. Behav. Exp. Finance* **9**, 88–97 (2016).

45. Simonsohn, U. Small Telescopes: Detectability and the Evaluation of Replication Results. *Psychol. Sci.* **26**, 559–569 (2015).

46. Ly, A., Verhagen, J. & Wagenmakers, E.-J. Harold Jeffreys's default Bayes factor hypothesis tests: Explanation, extension, and application in psychology. *J. Math. Psychol.* **72**, 19–32 (2016).

47. Ly, A., Etz, A., Marsman, M. & Wagenmakers, E.-J. Replication Bayes factors from evidence updating. *Behav. Res. Methods* **51**, 2498–2508 (2019).

48. Patil, P., Peng, R. D. & Leek, J. T. What should researchers expect when they replicate studies? A statistical view of replicability in psychological science. *Perspect. Psychol. Sci.* **11**, 539–544 (2016).

Supplementary Figures

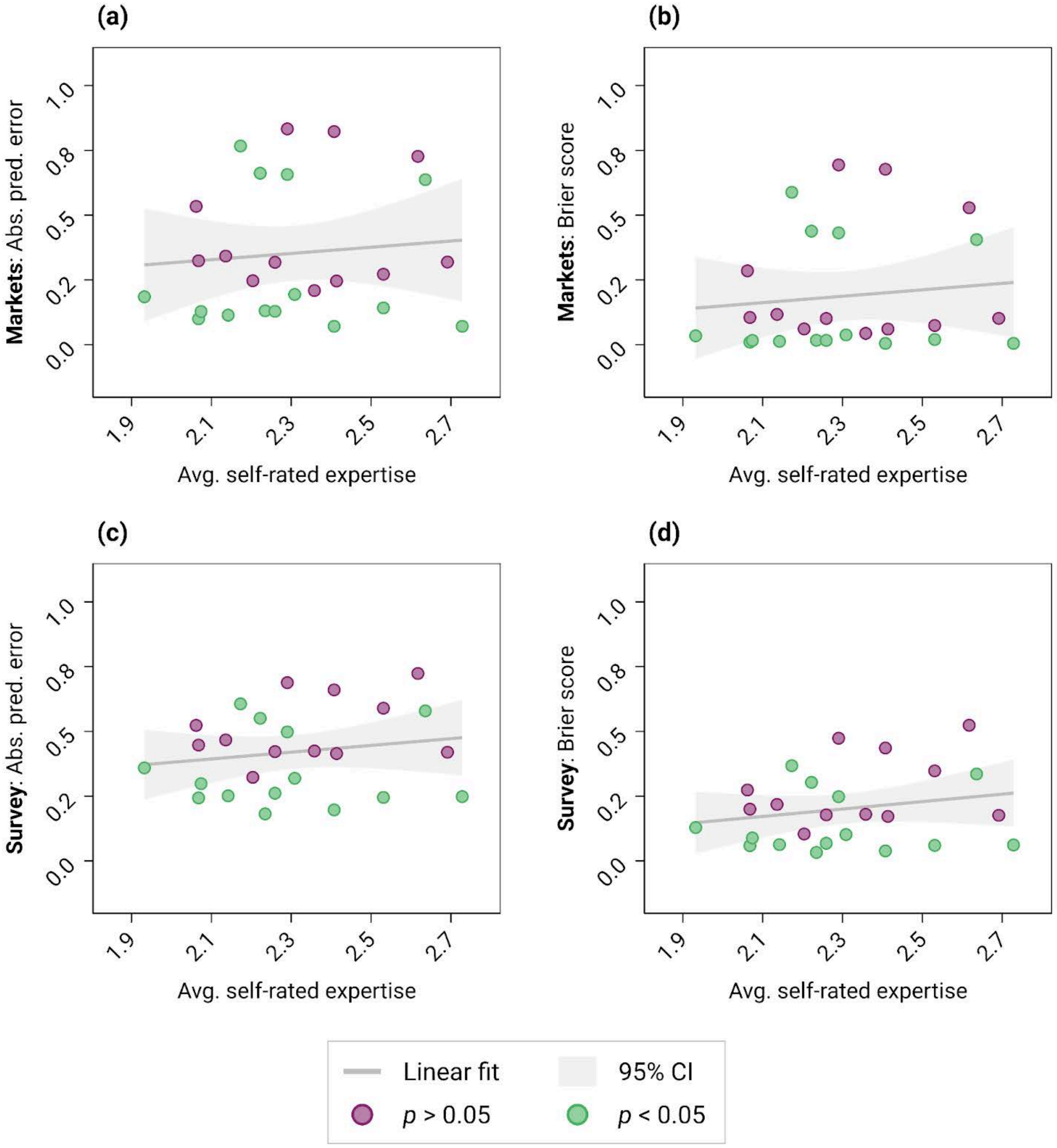

Supplementary Figure 1. Association between prediction accuracy and forecasters' average self-rated expertise (not preregistered). a, Plotted are the average self-rated expertise of the forecasters on a scale from 1 to 7 for each of the 26 replication studies and the absolute prediction error of the 26 decision market forecasts (Pearson's ρ = 0.100, 95% CI [−0.299, 0.469]; $t(24) = 0.490$, $p = 0.629$; $n = 26$). **b,** Plotted are the average self-rated expertise of the forecasters on a scale from 1 to 7 for each of the 26 replication studies and the Brier score (squared prediction error) of the 26 decision market forecasts (Pearson's ρ = 0.114, 95% CI [−0.286, 0.481]; $t(24) = 0.565$, $p = 0.577$; $n = 26$). **c,** Plotted are the average self-rated expertise of the forecasters on a scale from 1 to 7 for each of the 26 replication studies and the absolute prediction error of the 26 survey forecasts (Pearson's ρ = 0.175, 95% CI [−0.228, 0.526]; $t(24) = 0.869$, $p = 0.393$; $n = 26$). **d,** Plotted are the average self-rated expertise of the forecasters on a scale from 1 to 7 for each of the 26 replication studies and the Brier score (squared prediction error) of the survey forecasts (Pearson's ρ = 0.214, 95% CI [−0.189, 0.555]; $t(24) = 1.073$, $p = 0.294$; $n = 26$).

Supplementary Tables

Supplementary Table 1. Hypotheses. The table reports the hypotheses picked for potential replication for the 41 studies, as presented to forecasters in the prediction survey and the decision market. The rightmost column indicates whether the hypothesis was selected for replication and, if so, whether the hypothesis belongs to the "top-12" or "bottom-12" group (as based on the final decision market prices), or whether it has been selected at random.

Study	*Hypothesis*	*Selected*
Ames, D. L. & Fiske, S. T.[1]	Reading a vignette describing a nursing home employee who mixed up patients' medications intentionally results in an increased choice of the "blame" task relative to the choice frequency of the blame task of participants who read a vignette in which the medications were mixed up unintentionally. To evaluate this hypothesis, the authors perform a χ^2-test ($\chi^2(1) = 6.7$, $p = 0.009$); p. 3601.	☑ *top-12*
Atir, S. & Ferguson, M. J.[2]	Researchers referred to by surname are allocated a larger share of the award money compared to researchers who are referred to by their full name. To evaluate this hypothesis, the authors use a test of fixed effects in a mixed-effects model controlling for the specific proposal and researcher name: Researchers referred to by surname were also allocated a larger share of the award money by 6%: $F(1,551) = 4.61$, $p = 0.032$ (Fig. 3); p. 7281.	☑ *bottom-12*
Baldwin, M. & Lammers, J.[3]	Conservatives become more pro-environmental when appeals to address climate change are framed as past-focused comparisons. To evaluate this hypothesis, the authors perform a regression analysis, with focus on the interaction result between political orientation and condition (interaction $b = 0.008$, $p = 0.03$): "As expected, conservatives expressed less favorable attitudes in the future-focused comparison ($b = -0.015$, $p < 0.0001$), but this association was greatly attenuated in the past-focused comparison ($b = -0.007$, $p = 0.011$; interaction $b = 0.008$, $p = 0.03$)" (p. 14955). The replication focuses on the interaction effect of political orientation and the condition.	☑ *bottom-12*
Bear, A., Fortgang, R. G., Bronstein, M. V. & Cannon, T. D.[4]	People more prone to delusion-like ideation have an exaggerated bias in accurate prediction in shorter trials. To evaluate this hypothesis, the authors use a multilevel logistic model, with a focus on the interaction effect of delay with PDI ($b = -0.013$, $z = -2.94$, $p = 0.003$); p. 10793.	☒

cont'd on next page

Study	*Hypothesis*	*Selected*
Boswell, R. G., Sun, W., Suzuki, S. & Kober, H.[5]	Participants who receive training in Regulation of Craving (ROC-T) with negative strategies subsequently increase healthy food choices in the presence of tempting alternatives. The authors test the above hypothesis in an independent-samples *t*-test comparing the participants' change (posttest − pretest) in hypothetical healthy food choices between the negative ROC-T and the control condition. (independent-samples *t*-test (not assuming equal variances): negative ROC-T vs. control: $t(173.36) = 5.37$, $p < 0.001$); p. 12 in the SI.	☑ *top-12*
Caruso, E. M., Burns, Z. C. & Converse, B. A.[6]	People report that a killing during an armed robbery is an action with more willful, deliberate, and premeditated intent to kill if they watch a video of the action in slow motion compared to regular speed. To evaluate this hypothesis, the authors perform an ANOVA ($F(1, 405) = 10.80$, $p = 0.001$, $\eta^2_p = 0.026$); p. 9252.	☒
Casella, A., Kartik, N., Sanchez, L. & Turban, S.[7]	Senders send higher non-binding promises to send back money to receivers when there are two senders than when there is only one sender. The authors test the above hypothesis in an independent-samples *t*-test ($t(594) = 3.465$, $p = 0.000568$, reported as $p < 0.001$ in the paper; the exact *t*-value and *p*-value was calculated by the replicating team based on the original data uploaded to PNAS); p. 935.	☒
Chao, M.[8]	People donate less to a charity when offered a visible gift card for the donation compared to when the gift card is not visible. To evaluate this hypothesis, the author performs a *t*-test comparing the share of participants donating in the gift/no-image treatment versus the gift/image treatment ($p = 0.021$); p. 7305. We randomly picked the gift/no-image treatment as the comparison to the gift/image treatment.	☑ *bottom-12*
Cheon, B. K. & Hong, Y.-Y.[9]	Participants in the low SSES condition exhibit stronger implicit preferences for calorie-dense foods over fruits/vegetables compared with those in the high SSES condition. The paper only reports a 2 (SSES: low or high) $\times$ 2 (gender) ANOVA, with a significant main effect for SSES ($F(1, 79) = 4.92$, $p = 0.03$); p. 74. As the replication only focuses on the effect of the subjective socioeconomic status manipulation, but neither on the effect of gender nor the interaction of gender and SSES, the authors kindly provided us with the result of a direct comparison of the implicit preferences for calorie-dense foods over fruits/vegetables between the two SSES conditions ($n_1 = 87$, $n_2 = 80$) based on an independent-samples *t*-test: $t(165) = 2.07$, $p = 0.04$.	☑ *bottom-12*

cont'd on next page

Study	*Hypothesis*	*Selected*
Clarkson, J.J., Chambers, J. R. Hirt, E. R., Otto, A. S., Kardes, F. R. & Leone, C.[10]	Conservatives show greater self-control when told that free will beliefs enhance self-control, whereas liberals show greater self-control when told that free will beliefs inhibit self-control. The authors test the above hypothesis in a hierarchical regression, with political ideology (continuous, mean-centered) and free will theory (0, belief in freewill impedes self-control; 1, belief in freewill enhances self-control) and their interaction term as predictors (along with demographics) with a *t*-test of the interaction coefficient in the regression (Political Ideology $\times$ Freewill Theory interaction: $\beta = 0.68$, $t(126) = 3.25$, $p = 0.002$, $R^2 = 0.19$); p. 8251.	☒
Cooney, G., Gilbert, D. T. & Wilson, T. D.[11]	Participants predict that they would feel less bad not receiving the bonus under the fair procedure compared to not receiving the bonus under the unfair procedure. Participants are asked to predict how they would feel under four different scenarios: 2 (Outcome: receive bonus or no bonus) $\times$ 2 (Procedure: fair or unfair). To evaluate this hypothesis, the authors perform an *F*-test on the Outcome $\times$ Procedure interaction ($F(1,119) = 54.23$, $p < 0.001$, $\eta^2_p = 0.313$); p. 4 in SI.	☑ *top-12*
Côté, S., House, J. & Willer, R.[12]	High economic inequality (as compared to low economic inequality) reduces the generosity of high-income individuals (as compared to low-income individuals). The above hypothesis is tested using an OLS regression, interacting the high inequality treatment with a dummy for high-income participants. Specifically, we replicate the result on the interaction coefficient of Model 1 in Table 2 ($b = -0.08$, $se = 0.04$, t-value $= -2.15$); p. 15840.	☑ *bottom-12*
Flesch, T., Balaguer, J., Dekker, R., Nili, H. & Summerfield, C.[13]	Blocked training (where one of two task rules is held constant for large blocks of trials) leads to higher performance on a subsequent interleaved task (where the two task rules vary randomly from trial to trial) than interleaved training. The authors test the above hypothesis in an independent-samples *t*-test ($t(93) = 2.32$, $p = 0.023$); Fig. 2A and p. 10315.	☒
Genschow, O., Rigoni, D. & Brass, M.[14]	Participants who read a book passage that reduces belief in free will show a smaller correspondence bias than control participants who read a book passage unrelated to free will. The authors test the above hypothesis in an independent-samples *t*-test ($t(502) = 2.10$, $p = 0.036$, $d = 0.19$); p. 10073.	☑ *bottom-12*

cont'd on next page

Study	*Hypothesis*	*Selected*
Gheorghiu, A. I., Callan, M. J. & Skylark, W. J.[15]	Participants are more likely to select articles that are paired with photographs of researchers that are associated with high competence. To evaluate this hypothesis, the authors perform a mixed-effects regression as reported in the Table S16 in the SI appendix (the test statistic and *p*-value for this result are not reported in the main text). The *p*-value for this result is $p = 0.032$. The original study does not report a test statistic corresponding to the treatment effect. Based on the original data and analysis scripts that were kindly shared by the original authors, the corresponding test statistic is $t(404) = 2.151$ (using Satterthwaite's correction for degrees of freedom). This particular result was chosen since it was the key result in study 4.	☑ *bottom-12*
Guilbeault, D., Becker, J. & Centola, D.[16]	Partisan priming reduces trend accuracy in networks when predicting climate trends. Trend accuracy is defined as the fraction of participants in a treatment that predicted the correct trend in the data, and the trends are compared in round 3. The authors test this hypothesis in a Mann-Whitney-U test with each network of 40 individuals aggregated into one observation and 12 aggregated observations per treatment (Mann-Whitney-U test, $n = 24$, $z = 3.207$, $p = 0.0013$; p. 9716). This test was randomly chosen.	☑ *random*
Halevy, N. & Halali, E.[17]	Intervening in a conflict between two friends is seen as more beneficial if individuals are asked to recall and describe a situation where they did intervene compared to when they are asked to recall and describe a situation where they did not intervene. The authors test the above hypothesis in an independent-samples *t*-test ($t(196) = 6.34$, $p = 0.000000001549$); p. 6940. This test was randomly picked among the main tests in experiment 6.	☑ *top-12*
Handley, I. M., Brown, E. R., Moss-Racusin, C. A. & Smith, J. L.[18]	Men evaluate an abstract reporting that gender-bias exists less favorably than women and an abstract reporting that no gender-bias exists more favorably than women. To evaluate this hypothesis, the authors perform an *F*-test on the gender × abstract type interaction ($F(1,299) = 4.00$, $p = 0.046$, $\eta^2_p = 0.013$); p. 13203.	☒

cont'd on next page

Study	*Hypothesis*	*Selected*
Hoffman, K. M., Trawalter, S., Axt, J. R. & Oliver, M. N.[19]	Non-medically trained, white individuals exhibit a racial bias in pain perception and this bias is related to holding false medical beliefs about blacks vs. whites. The authors test this hypothesis by aggregating the Likert scale surveys continuously and regressing pain ratings on target race, false beliefs, and their interaction, controlling for age, gender, and self-ratings of pain. The authors report a significant and negative interaction between target race and false beliefs, $\beta = -0.07$, $se = 0.03$, $F(1,85) = 4.36$, p = 0.040, $\eta^2_p = 0.05$); p. 4297.	☑ *bottom-12*
Hofstetter, R., Rüppell, R. & John, L. K.[20]	Temporariness decreases privacy concerns about sharing a selfie. To evaluate this hypothesis, the authors perform an ordinary least squares regression of subjects' privacy concerns on an indicator variable for the "temporary" condition; $\beta = -0.70$, $se = 0.22$, $p < 0.005$; p. 11903.	☑ *top-12*
Horne, Z., Powell, D., Hummel, J. E. & Holyoak, K. J.[21]	Participants who are exposed to factual information about the dangers of communicable diseases report a larger positive change in attitudes towards vaccines than participants in an alternative intervention aimed at undercutting vaccination myths. After an initial ANOVA including all three treatments, the above hypothesis is tested in an independent-samples *t*-test comparing the participants' change (posttest−pretest) in vaccine attitude scores between the disease risk treatment and the autism correction treatment (independent-samples *t*-test assuming equal variances: $t(203) = 2.41$, $p = 0.017$); p. 10322. This test was randomly chosen.	☒
Isley, S. C., Stern, P. C., Carmichael, S. P., Joseph, K. M. & Arent, D. J.[22]	Indicating the environmental friendliness of an Airbnb rental through a leaf increases willingness to pay compared to having no leaf on the rental. The authors test the above hypothesis with a *z*-test ($z = 3.05$, $p = 0.002$); p. 9783.	☒
Jachimowicz, J. M., Chafik, S., Munrat, S., Prabhu, J. C. & Weber, E. U.[23]	Low felt-income individuals with low levels of community trust are more myopic than low felt-income individuals with high levels of community trust. The authors test the above hypothesis using a two-sided independent-samples *t*-test ($t(60) = 2.79$, $p = 0.007$; since the article did not include test statistics, the results were re-estimated based on the original data and analysis scripts provided by the authors. Note that the re-estimated *p*-value deviates from the *p*-value mentioned in the article ($p = 0.04$); p. 5404).	☒

cont'd on next page

Study	*Hypothesis*	*Selected*
John, L. K., Barasz, K. & Norton, M. I.[24]	People are more interested in potential dates who inadvertently did not answer all questions on their desirable behaviors than potential dates who deliberately do not provide answers to all questions. To evaluate this hypothesis, the authors perform an independent-samples *t*-test ($t(140) = 2.08$, $p = 0.04$); p. 956.	☑ *bottom-12*
Jordan, J. J., Hoffman, M., Nowak, M. A. & Rand, D. G.[25]	Participants are more likely to behave in an uncalculating manner in the process observable condition (when their reputation is at stake) than in the process hidden condition (when their reputation is not at stake). To evaluate this hypothesis, the authors perform a logistic regression ($b = -0.486$, $p = 0.002$); p. 8660.	☑ *random*
Jun, Y., Meng, R. & Johar, G. V.[26]	People flag more for false news when alone than in the presence of others. To evaluate this hypothesis, the authors perform an *F*-test ($F(1,284) = 7.07$, $p = 0.01$); *p*. 5979. We randomly picked “large” as the group size to compare against “alone.” As the replication only focuses on two of the three conditions, we replace the *F*-test result by an independent-samples *t*-test, based on the means and standard deviations reported in the paper (assuming $n_1 = n_2 = 96$): $t(190) = 2.555$, $p = 0.011$.	☒
KC, R. P., Kunter, M. & Mak, V.[27]	As the competition reward increases from zero to a low level, social comparison motivation increases among non-competitors and therefore their performance increases. The authors test the above hypothesis in an independent-samples *t*-test ($t(169) = 2.37$, $p = 0.019$); p. 2719. This test was randomly picked among the main tests in the competition treatments.	☒
Klein, N. & O’Brien, E.[28]	Participants expect that they need to see a higher number of paintings in order to reach a verdict about whether or not they like a painting style compared to the number that participants experiencing the paintings actually see before reaching a verdict. To evaluate this hypothesis, the authors perform a *t*-test ($t(205) = 10.60$, $p < 0.001$); p. 13223.	☑ *top-12*
Kouchaki, M. & Gino, F.[29]	The possibility to cheat in a die-throwing game results in more dishonesty in a Boggle game after three days. The hypothesis is evaluated using a χ^2-test between the percentages of cheating participants in the likely cheating treatment vs. the no-cheating treatment ($\chi^2(1) = 10.48$, $p = 0.001$, $n = 258$, Cramer’s $V = 0.20$); p. 6170.	☑ *bottom-12*

cont’d on next page

Study	*Hypothesis*	*Selected*
Kraus, M. W., Rucker, J. M. & Richeson, J. A.[30]	Inducing consideration of the persistence of racial discrimination in the United States reduces the overestimation of racial economic equality. To evaluate this hypothesis, the authors compare the overestimates of racial economic equality in the "Discriminatory USA" treatments with the overestimates of racial economic equality in the "Current" treatment (i.e., the participants' current perception of racial economic equality). The authors test the above hypothesis using a paired t-test, $t(201) = 17.51$, $p < 0.001$, $d_{RM} = 0.50$; p. 10329. Note, however, the test could not be replicated using the original dataset. When testing estimates of equality of "Discriminatory USA" and "Current" using a paired t-test, the test statistic we calculate is $t(201) = 7.0258$, p < 0.001. The reporting error in the original paper has also been acknowledged by the original authors. We thus base our power analysis on the latter test statistic.	☑ *top-12*
McCall, L., Burk, D., Laperrière, M. & Richeson, J. A.[31]	Participants exposed to inequality compared to a control are more likely to rate structural factors as more important in "getting ahead." To evaluate this hypothesis, the authors perform a regression analysis ($\beta = 0.353$, $se = 0.076$, $p < 0.001$, root-mean-squared error (RMSE) = 0.828 for "structural factors"); p. 9594. We randomly picked the "structural factors" result.	☑ *top-12*
Morris, A., MacGlashan, J., Littman, M. L. & Cushman, F.[32]	In repeated games against rigid opponents, participants assigned to be thieves act relatively flexibly in their stealing behavior while participants assigned to be victims act relatively rigidly in their punishment behavior. The authors test the above hypothesis through an interaction between role and round in a mixed-effects model using a likelihood ratio test ($\chi^2(1) = 24.3$, $p < 0.001$); p. 10399.	☑ *top-12*
Mummolo, J.[33]	When participants in a mock news article with an unnamed police chief seeking a budget increase are exposed to an image of high militarization, support for police funding in the United States falls compared to when they view an image with traditional police. The treatment effect was estimated via an OLS regression with robust "HC1" standard errors, where the police funding outcome was regressed on an intercept and indicators for all treatment conditions with the control condition excluded as the baseline. The outcome was measured on a 5-point scale but rescaled to range between 0 and 1. The effect estimate was −0.036902 ($p = 0.0204$). This particular result was chosen because it was a key result of this experiment.	☒

cont'd on next page

Study	*Hypothesis*	*Selected*
Payne, B. K., Brown-Iannuzzi, J. L. & Hannay, J. W.[34]	High inequality leads to higher risk-taking than low inequality. To evaluate this hypothesis, the authors perform an independent-samples t-test on the average probability of getting the low outcome across all three gambles ($t(219) = 2.21$, $p = 0.028$); p. 4644. This test was chosen since it was the key result in study 1.	☒
Phillips, J. & Cushman, F.[35]	Participants judge immoral events as more impossible when they are not able to deliberate compared to after deliberating. To evaluate this hypothesis, the authors perform a *z*-test ($z = 5.423$, $p < 0.001$); p. 4650. This particular test was chosen since it was the key result in study 1a.	☒
Rai, T. S., Valdesolo, P. & Graham, J.[36]	People are more willing to harm a stranger when he has been described in dehumanized terms rather than humanized terms when the motives are instrumental. To evaluate this hypothesis, the authors perform a t-test comparing the share of participants reporting that they would break the stranger's thumb for money in dehumanized treatment versus the humanized treatment. The willingness to harm a stranger is higher in the dehumanized treatment compared to the humanized treatment ($t = 2.26$, $p = 0.025$); p. 8513.	☑ *bottom-12*
Reeck, C., Wall, D. & Johnson, E. J.[37]	Comparative searchers make more patient choices (i.e., choose a larger monetary reward which is delivered later) than integrative searchers. The authors test the above hypothesis in a hierarchical logistic regression ($\beta = -0.23$, $se = 0.10$, $p = 0.027$); pp. 11892–11893.	☑ *bottom-12*
Schilke, O., Reimann, M. & Cook, K. S.[38]	People with high structural power are less trusting than those with low structural power. Structural power is manipulated by participants imaging being a skilled typist who has either a high financial need to sell their service while getting only a few jobs offered (low power) vs. a low financial need to sell their service while getting many jobs offered (high power). Trust is measured by whether or not participants choose to give a free sample (i.e., trust the client to come back with a follow-up job). It is hypothesized that participants in the high (vs. low) power condition are less willing to provide a free sample. To evaluate this hypothesis, the authors conduct a χ^2-test ($\chi^2(1) = 18.23$, $p < 0.001$); p. 12952.	☒
Stern, C., West, T. V. & Rule, N. O.[39]	Conservatives allocate less money to targets who deviate from stereotypes (i.e., counterstereotypical targets). To evaluate this hypothesis, the authors perform a generalized estimating equations regression analysis ($b = 0.52$, $se = 0.14$, $z = 3.65$, $p < 0.001$); p. 15339.	☑ *top-12*

cont'd on next page

Study	Hypothesis	Selected
Vacharkulksemsuk, T., Reit, E., Khambatta, P., Eastwick, P. W., Finkel, E. J. & Carney, D. R.[40]	People rate men and women in expansive postures in photographs as more dominant than men and women in contracted postures photographs. To evaluate this hypothesis, the authors perform an independent-samples *t*-test; $t(424) = 18.02$, $p < 0.0001$, 95% CI (1.19, 1.48); p. 4012.	☑ *top-12*
Williams, K. E. G., Sng, O. & Neuberg, S. L.[41]	High-wealth individuals from desperate ecologies are stereotyped as possessing faster life history strategies (e.g., act impulsively, have more children than can be financially supported) than high-wealth individuals from hopeful ecologies. To evaluate this hypothesis, the authors perform independent-samples *t*-tests for each of five life history strategy-relevant suites separately: sexual unrestrictedness, impulsivity, opportunistic behavior, investment in own education, and investment in children. We instead create a composite score of all five life history strategies using the original data and perform an independent sample Student's *t*-test ($t(94) = 4.66$, $p < .001$). This test statistic has been re-estimated based on the original data. The high wealth condition was randomly chosen. [Note: in the original paper, the ecology effect is tested for all five life history strategy-relevant suites separately between a desperate or a hopeful ecology. The differences were statistically significant $p < 0.05$, with effect sizes ranging from 0.42 to 1.21 (the test used was not reported, but we assume an independent sample *t*-test was used); p. 6 in SI.]	☑ *top-12*

Supplementary Table 2. Decision market results. The table provides descriptive statistics on the number of trades (i.e., transactions), the number of traders, the average number of tokens invested per transaction (the initial endowment was 100 tokens per trader), and the average number of shares bought or sold per hypothesis, alongside the final decision market price and a column indicating whether the study was selected for replication (and, if so, whether the hypothesis belongs to the "top-12" or "bottom-12" group (as based on the final decision market prices), or whether it has been selected at random). Furthermore, the absolute prediction error and the Brier score (i.e., the squared prediction error) pertaining to the decision market forecasts are reported beside an indicator for whether the replication was successful (as per the statistical significance indicator).

Study	*Total # trades*	*Total # traders*	*Avg. # tokens invested*	*Avg. # shares transacted*	*Decision market price*	*Selected*	*Replication outcome*	*Absolute prediction error*	*Brier score*
Ames & Fiske[1]	126	79	11.6	5.7	0.833	top-12	☒	0.833	0.694
Atir & Ferguson[2]	171	95	13.1	7.0	0.209	bottom-12	☒	0.209	0.044
Baldwin & Lammers[3]	123	74	7.1	3.6	0.246	bottom-12	☒	0.246	0.061
Bear et al.[4]	72	48	7.9	3.9	0.570	—	☐	—	—
Boswell et al.[5]	102	69	9.5	5.1	0.886	top-12	☑	0.114	0.013
Caruso et al.[6]	106	65	9.4	4.7	0.757	—	☐	—	—
Casella et al.[7]	103	65	6.9	3.7	0.796	—	☐	—	—
Chao[8]	112	75	7.4	3.8	0.363	bottom-12	☑	0.637	0.406
Cheon & Hong[9]	166	90	8.4	4.2	0.247	bottom-12	☒	0.247	0.061
Clarkson et al.[10]	112	61	8.7	4.3	0.464	—	☐	—	—
Cooney et al.[11]	150	86	7.8	4.3	0.929	top-12	☑	0.071	0.005
Côté et al.[12]	137	75	9.3	4.6	0.319	bottom-12	☒	0.319	0.102
Flesch et al.[13]	59	35	4.6	2.3	0.533	—	☐	—	—
Genschow et al.[14]	159	87	11.4	5.0	0.233	bottom-12	☑	0.767	0.588

cont'd on next page

Study	*Total # trades*	*Total # traders*	*Avg. # tokens invested*	*Avg. # shares transacted*	*Decision market price*	*Selected*	*Replication outcome*	*Absolute prediction error*	*Brier score*
Gheorghiu et al.[15]	98	68	8.0	4.1	0.338	bottom-12	☑	0.662	0.438
Guilbeault et al.[16]	87	59	12.1	6.1	0.534	random	☒	0.534	0.285
Halevy & Halali[17]	120	80	8.5	4.8	0.900	top-12	☑	0.100	0.010
Handley et al.[18]	115	62	14.4	7.3	0.481	—	☐	—	—
Hoffman et al.[19]	115	83	9.2	4.5	0.318	bottom-12	☒	0.318	0.101
Hofstetter et al.[20]	79	58	8.8	4.4	0.872	top-12	☑	0.128	0.016
Horne et al.[21]	62	43	7.1	3.6	0.563	—	☐	—	—
Isley et al.[22]	63	51	9.0	4.5	0.588	—	☐	—	—
Jachimowicz et al.[23]	121	61	9.9	5.0	0.585	—	☐	—	—
John et al.[24]	117	67	7.8	3.9	0.324	bottom-12	☒	0.324	0.105
Jordan et al.[25]	98	54	10.1	5.2	0.727	random	☒	0.727	0.529
Jun et al.[26]	56	41	6.0	3.0	0.468	—	☐	—	—
KC et al.[27]	58	39	6.2	3.1	0.440	—	☐	—	—
Klein & O'Brien[28]	142	79	8.1	4.4	0.869	top-12	☑	0.131	0.017
Kouchaki & Gino[29]	110	61	9.3	4.7	0.272	bottom-12	☒	0.272	0.074
Kraus et al.[30]	85	50	7.7	4.0	0.806	top-12	☑	0.194	0.038
McCall et al.[31]	104	68	8.1	4.3	0.858	top-12	☑	0.142	0.020
Morris et al.[32]	88	61	8.3	4.6	0.871	top-12	☑	0.871	0.759
Mummolo[33]	86	58	8.9	4.4	0.502	—	☐	—	—

cont'd on next page

Study	*Total # trades*	*Total # traders*	*Avg. # tokens invested*	*Avg. # shares transacted*	*Decision market price*	*Selected*	*Replication outcome*	*Absolute prediction error*	*Brier score*
Payne et al.[34]	65	44	7.3	3.6	0.466	—	☐	—	—
Phillips & Cushman[35]	168	81	14.2	7.3	0.448	—	☐	—	—
Rai et al.[36]	96	62	8.7	4.2	0.343	bottom-12	☑	0.657	0.432
Reeck et al.[37]	71	51	7.8	3.7	0.342	bottom-12	☒	0.342	0.117
Schilke et al.[38]	85	60	6.4	3.6	0.766	—	☐	—	—
Stern et al.[39]	117	64	9.0	4.8	0.823	top-12	☒	0.823	0.677
Vacharkulksemsuk et al.[40]	213	98	35.8	8.2	0.929	top-12	☑	0.071	0.005
Williams et al.[41]	95	64	9.6	5.1	0.815	top-12	☑	0.185	0.034

Supplementary Table 3. Original and replication results. The table shows the results of the 26 replications alongside the corresponding original results. *n* indicates the sample size, *d* denotes the standardized effect size (in Cohen's *d* units), 95% CI denotes the 95% confidence interval around *d*, and *p* indicates the (two-sided) *p*-value. The effect sizes of the original studies are set to positive values; for the replications, positive effect sizes (*d*) correspond to effects in the same direction as in the original study whereas negative values indicate results in the opposite direction. The planned replication sample sizes were set to the maximum of the sample size allowing for detection of ⅔ of the original effect size with 90% probability and the original sample size. *Power* indicates the (ex ante) statistical power of the replication to detect ⅔ of the original effect size for the eventual number of observations collected. The two rightmost columns tabulate our two primary replication indicators: the statistical significance indicator (i.e., an effect in the same direction as in the original study with $p < 0.05$) and the relative effect size (i.e., the ratio of the replication effect size to the original effect size). We followed the original article as closely as possible in terms of the statistical analyses and used the same statistical test in the replication study as in the original study whenever possible; see the Methods section and the replication reports (https://osf.io/sejyp) for the exact statistical test used in the original studies and the replication studies.

	Original study				*Replication study*						
Study	*n*	*d*	*95% CI*	*p*	*n*	*d*	*95% CI*	*p*	*Power*	*Relative effect size*	*Stat. sig. criterion*
Ames & Fiske[1]	201	0.365	[0.089, 0.642]	0.010	723	0.027	[−0.119, 0.173]	0.714	0.905	0.075	☒
Atir & Ferguson[2]	554	0.091	[0.008, 0.175]	0.032	2,849	0.009	[−0.028, 0.046]	0.639	0.901	0.096	☒
Baldwin & Lammers[3]	200	0.628	[0.070, 1.186]	0.028	999	0.049	[−0.199, 0.297]	0.699	0.911	0.078	☒
Boswell et al.[5]	260	0.666	[0.421, 0.911]	$2.5e^{-07}$	291	0.829	[0.598, 1.061]	$5.7e^{-11}$	0.966	1.245	☑
Chao[8]	519	0.204	[0.032, 0.377]	0.020	2,441	0.104	[0.025, 0.184]	0.010	0.920	0.510	☑
Cheon & Hong[9]	167	0.320	[0.015, 0.626]	0.040	925	−0.054	[−0.183, 0.075]	0.408	0.901	−0.170	☒
Cooney et al.[11]	120	0.672	[0.491, 0.853]	$2.5e^{-11}$	132	0.244	[0.071, 0.416]	0.006	0.999	0.362	☑
Côté et al.[12]	704	0.324	[0.028, 0.620]	0.032	3,653	−0.013	[−0.143, 0.116]	0.841	0.904	−0.041	☒
Genschow et al.[14]	504	0.187	[0.012, 0.362]	0.036	2,822	0.138	[0.064, 0.211]	$2.6e^{-04}$	0.912	0.735	☑
Gheorghiu et al.[15]	408	0.106	[0.009, 0.204]	0.032	2,213	0.145	[0.103, 0.187]	$8.9e^{-12}$	0.916	1.362	☑

cont'd on next page

	Original study				*Replication study*						
Study	*n*	*d*	*95% CI*	*p*	*n*	*d*	*95% CI*	*p*	*Power*	*Relative effect size*	*Stat. sig. criterion*
Guilbeault et al.[16]	24	1.309	[0.509, 2.109]	0.001	56	−0.068	[−0.592, 0.456]	0.799	0.904	−0.052	☒
Halevy & Halali[17]	198	0.901	[0.621, 1.181]	$1.5e^{-09}$	227	0.599	[0.337, 0.860]	$1.0e^{-05}$	0.995	0.665	☑
Hoffman et al.[19]	92	0.871	[0.042, 1.700]	0.040	503	0.092	[−0.258, 0.443]	0.605	0.902	0.106	☒
Hofstetter et al.[20]	323	0.347	[0.128, 0.566]	0.002	820	0.332	[0.195, 0.469]	$2.4e^{-06}$	0.912	0.956	☑
John et al.[24]	142	0.349	[0.017, 0.681]	0.039	1,224	0.039	[−0.073, 0.151]	0.492	0.983	0.113	☒
Jordan et al.[25]	735	0.230	[0.085, 0.374]	0.002	1,826	0.050	[−0.042, 0.141]	0.288	0.906	0.216	☒
Klein & O'Brien[28]	207	1.474	[1.199, 1.748]	$3.3e^{-21}$	214	1.386	[1.116, 1.655]	$6.2e^{-20}$	> 0.999	0.940	☑
Kouchaki & Gino[29]	258	0.403	[0.159, 0.647]	0.001	584	−0.038	[−0.200, 0.124]	0.646	0.901	−0.094	☒
Kraus et al.[30]	202	0.494	[0.356, 0.633]	$3.2e^{-11}$	205	0.259	[0.121, 0.397]	$2.7e^{-04}$	0.997	0.524	☑
McCall et al.[31]	480	0.423	[0.243, 0.602]	$4.7e^{-06}$	679	0.188	[0.037, 0.338]	0.015	0.957	0.444	☑
Morris et al.[32]	100	0.721	[0.329, 1.113]	$3.2e^{-04}$	128	0.414	[0.068, 0.761]	0.019	0.776	0.575	☑
Rai et al.[36]	182	0.335	[0.043, 0.628]	0.025	855	0.185	[0.051, 0.320]	0.007	0.904	0.554	☑
Reeck et al.[37]	207	0.307	[0.035, 0.579]	0.027	1,044	0.067	[−0.054, 0.188]	0.278	0.911	0.219	☒
Stern et al.[39]	273	0.221	[0.102, 0.340]	$2.6e^{-04}$	503	0.011	[−0.076, 0.099]	0.801	0.910	0.051	☒
Vacharkulksemsuk et al.[40]	426	1.746	[1.556, 1.937]	$2.6e^{-54}$	450	0.974	[0.789, 1.160]	$1.4e^{-22}$	> 0.999	0.558	☑
Williams et al.[41]	96	0.951	[0.546, 1.357]	$1.0e^{-05}$	112	0.621	[0.246, 0.995]	0.001	0.919	0.653	☑

Supplementary Table 4. Results pertaining to the secondary replication indicators. For each of the 26 replications, the table reports *(i)* the fixed-effects weighted meta-analytic effect size (in Cohen's *d* units) and its 95% confidence interval and the corresponding two-sided *p*-values based on a z-test alongside indicators for the statistical significance indicator (i.e., effect in the same direction as in the original study with $p < \alpha$), evaluated at $\alpha = 0.05$ and $\alpha = 0.005$, respectively; *(ii)* the effect size the original study could detect with 33% power at the 5% significance level ("small effect;" $d_{0.33}$) alongside the "small telescopes" replication indicator (i.e., whether the replication effect size is not significantly smaller than the "small effect" in a one-sided test at the 5% significance level)[45]; and *(iii)* the one-sided default Bayes factor (BF_{+0})[46] and the one-sided replication Bayes factor (BF_{R0})[47].

	Meta-analytic effect					Small telescopes		Bayes factors	
Study	*d*	*95% CI*	*p*	*Stat. sig. at 5%*	*Stat. sig. at 0.5%*	$d_{0.33}$	*Replication indicator*	BF_{+0}	BF_{R0}
Ames & Fiske[1]	0.101	[−0.028, 0.230]	0.126	☒	☒	0.216	☒	0.058	0.053
Atir & Ferguson[2]	0.022	[−0.011, 0.056]	0.195	☒	☒	0.065	☒	0.040	0.118
Baldwin & Lammers[3]	0.145	[−0.081, 0.372]	0.208	☒	☒	0.436	☒	0.099	0.083
Boswell et al.[5]	0.752	[0.585, 0.919]	$1.1e^{-18}$	☑	☑	0.191	☑	$1.3e^{+09}$	$4.0e^{+09}$
Chao[8]	0.122	[0.050, 0.194]	0.001	☑	☑	0.135	☑	2.437	7.219
Cheon & Hong[9]	0.003	[−0.116, 0.122]	0.962	☒	☒	0.239	☒	0.042	0.030
Cooney et al.[11]	0.448	[0.324, 0.571]	$1.2e^{-12}$	☑	☑	0.141	☑	8.015	0.235
Côté et al.[12]	0.041	[−0.078, 0.160]	0.496	☒	☒	0.231	☒	0.032	0.041
Genschow et al.[14]	0.145	[0.077, 0.213]	$2.9e^{-05}$	☑	☑	0.137	☑	63.954	282.877
Gheorghiu et al.[15]	0.139	[0.101, 0.177]	$1.1e^{-12}$	☑	☑	0.076	☑	$5.9e^{+08}$	$3.9e^{+09}$
Guilbeault et al.[16]	0.345	[−0.093, 0.784]	0.123	☒	☒	0.624	☒	0.227	0.029
Halevy & Halali[17]	0.740	[0.550, 0.930]	$2.5e^{-14}$	☑	☑	0.219	☑	$3.1e^{+03}$	$4.7e^{+03}$
Hoffman et al.[19]	0.213	[−0.109, 0.534]	0.195	☒	☒	0.649	☒	0.157	0.128

cont'd on next page

	Meta-analytic effect					Small telescopes		Bayes factors	
Study	*d*	*95% CI*	*p*	*Stat. sig. at 5%*	*Stat. sig. at 0.5%*	$d_{0.33}$	*Replication indicator*	BF_{+0}	BF_{R0}
Hofstetter et al.[20]	0.336	[0.220, 0.452]	$1.3e^{-08}$	☑	☑	0.171	☑	$9.0e^{+03}$	$3.7e^{+04}$
John et al.[24]	0.072	[−0.035, 0.178]	0.186	☒	☒	0.259	☒	0.122	0.102
Jordan et al.[25]	0.101	[0.024, 0.179]	0.010	☑	☒	0.113	☑	0.157	0.122
Klein & O'Brien[28]	1.429	[1.238, 1.620]	$1.2e^{-48}$	☑	☑	0.214	☑	$1.2e^{+17}$	$1.1e^{+18}$
Kouchaki & Gino[29]	0.097	[−0.038, 0.232]	0.159	☒	☒	0.190	☒	0.033	0.008
Kraus et al.[30]	0.376	[0.279, 0.473]	$3.4e^{-14}$	☑	☑	0.108	☑	110.289	45.719
McCall et al.[31]	0.285	[0.170, 0.400]	$1.2e^{-06}$	☑	☑	0.140	☑	3.125	2.131
Morris et al.[32]	0.549	[0.289, 0.808]	$3.4e^{-05}$	☑	☑	0.306	☑	2.809	5.859
Rai et al.[36]	0.212	[0.090, 0.333]	0.001	☑	☑	0.229	☑	5.581	12.334
Reeck et al.[37]	0.107	[−0.004, 0.218]	0.059	☒	☒	0.213	☒	0.108	0.218
Stern et al.[39]	0.085	[0.015, 0.155]	0.018	☑	☒	0.093	☒	0.062	0.013
Vacharkulksemsuk et al.[40]	1.350	[1.217, 1.482]	$9.4e^{-89}$	☑	☑	0.149	☑	$5.2e^{+19}$	$1.4e^{+15}$
Williams et al.[41]	0.773	[0.502, 1.045]	$2.4e^{-08}$	☑	☑	0.317	☑	44.426	76.618

Supplementary Table 5. Prediction intervals (not preregistered). The table shows the effect sizes (Cohen's *d*) and corresponding standard errors of the original (d_O, se_O) and the replication studies (d_R, se_R), the 95% prediction intervals[48] alongside a dichotomous variable indicating whether or not the replication effect (d_R) size falls into the 95% prediction interval, and the results of a two-sided *z*-test comparing the effect sizes of the replication and original studies. The effect sizes of the original studies are set to positive values; for the replications, positive effect sizes correspond to effects in the same direction as in the original study whereas negative values indicate results in the opposite direction. * $p < 0.05$, ** $p < 0.005$.

	Original study		*Replication study*		*Prediction interval*		*z-Test: $d_R = d_O$*	
Study	d_O	se_O	d_R	se_R	*95% PI*	*Replicated?*	*z*	*p*
Ames & Fiske[1]	0.365	0.141	0.027	0.074	[0.053, 0.678]	☒	−2.119	0.034*
Atir & Ferguson[2]	0.091	0.042	0.009	0.019	[0.000, 0.182]	☑	−1.775	0.076
Baldwin & Lammers[3]	0.628	0.283	0.049	0.127	[0.021, 1.235]	☑	−1.869	0.062
Boswell et al.[5]	0.666	0.124	0.829	0.117	[0.332, 1.001]	☑	0.957	0.339
Chao[8]	0.204	0.088	0.104	0.04	[0.015, 0.394]	☑	−1.034	0.301
Cheon & Hong[9]	0.320	0.155	−0.054	0.066	[−0.009, 0.650]	☒	−2.229	0.026*
Cooney et al.[11]	0.672	0.091	0.244	0.087	[0.425, 0.919]	☒	−3.399	0.001**
Côté et al.[12]	0.324	0.151	−0.013	0.066	[0.001, 0.647]	☒	−2.049	0.040*
Genschow et al.[14]	0.187	0.089	0.138	0.038	[−0.002, 0.377]	☑	−0.512	0.609
Gheorghiu et al.[15]	0.106	0.050	0.145	0.021	[0.001, 0.212]	☑	0.715	0.474
Guilbeault et al.[16]	1.309	0.408	−0.068	0.267	[0.353, 2.266]	☒	−2.822	0.005**
Halevy & Halali[17]	0.901	0.142	0.599	0.133	[0.520, 1.282]	☑	−1.554	0.120
Hoffman et al.[19]	0.871	0.417	0.092	0.178	[−0.018, 1.760]	☑	−1.716	0.086
Hofstetter et al.[20]	0.347	0.111	0.332	0.070	[0.090, 0.605]	☑	−0.117	0.907
John et al.[24]	0.349	0.168	0.039	0.057	[0.002, 0.697]	☑	−1.747	0.081

cont'd on next page

	Original study		*Replication study*		*Prediction interval*		*z-Test: $d_R = d_O$*	
Study	d_O	se_O	d_R	se_R	*95% PI*	*Replicated?*	z	p
Jordan et al.[25]	0.230	0.074	0.050	0.047	[0.059, 0.401]	☒	−2.062	0.039*
Klein & O'Brien[28]	1.474	0.139	1.386	0.137	[1.091, 1.856]	☑	−0.451	0.652
Kouchaki & Gino[29]	0.403	0.125	−0.038	0.083	[0.110, 0.696]	☒	−2.951	0.003**
Kraus et al.[30]	0.494	0.070	0.259	0.070	[0.300, 0.689]	☒	−2.375	0.018*
McCall et al.[31]	0.423	0.091	0.188	0.077	[0.189, 0.657]	☒	−1.973	0.049*
Morris et al.[32]	0.721	0.200	0.414	0.177	[0.197, 1.244]	☑	−1.147	0.251
Rai et al.[36]	0.335	0.148	0.185	0.068	[0.015, 0.655]	☑	−0.916	0.360
Reeck et al.[37]	0.307	0.139	0.067	0.062	[0.009, 0.605]	☑	−1.577	0.115
Stern et al.[39]	0.221	0.061	0.011	0.045	[0.074, 0.368]	☒	−2.789	0.005*
Vacharkulksemsuk et al.[40]	1.746	0.097	0.974	0.094	[1.481, 2.011]	☒	−5.709	$1.1e^{-08}$**
Williams et al.[41]	0.951	0.204	0.621	0.189	[0.406, 1.496]	☑	−1.187	0.235

Supplementary Table 6. Correlations between replication indicators (not preregistered). The table reports Kendall's rank correlation coefficients τ_b between the various replication indicators reported in the main text. *p*-values are reported in parentheses. Dichotomous replication indicators are marked with a dagger (†), continuous indicators are marked with a double dagger (‡). $n = 26$ for all correlations. * $p < 0.05$, ** $p < 0.005$; two-sided tests.

	Stat. sig. indicator†	*Relative effect size*‡	*Small telescopes*†	*Meta effect α = 5%*†	*Meta effect α = 0.5%*†	BF_{+0}‡	BF_{R0}‡	*Prediction intervals*†	$p(d_R - d_O)$‡
Statistical significance indicator†	1.000<br>—								
Relative effect size‡	0.719**<br>(p = 1.7e^{-05})	1.000<br>—							
Small-telescopes approach†	0.925**<br>(p = 4.3e^{-06})	0.704**<br>(p = 2.6e^{-05})	1.000<br>—						
Meta-analytic effect (α = 5%)†	0.854**<br>(p = 2.2e^{-05})	0.640**<br>(p = 1.3e^{-04})	0.923**<br>(p = 4.5e^{-06})	1.000<br>—					
Meta-analytic effect (α = 0.5%)†	1.000**<br>(p = 6.7e^{-07})	0.719**<br>(p = 1.7e^{-05})	0.925**<br>(p = 4.3e^{-06})	0.854**<br>(p = 2.2e^{-05})	1.000<br>—				
Default Bayes factor (BF_{+0})‡	0.719**<br>(p = 1.7e^{-05})	0.723**<br>(p = 2.5e^{-07})	0.704**<br>(p = 2.6e^{-05})	0.649**<br>(p = 1.1e^{-04})	0.719**<br>(p = 1.7e^{-05})	1.000<br>—			
Replication Bayes factor (BF_{R0})‡	0.719**<br>(p = 1.7e^{-05})	0.852**<br>(p = 1.2e^{-09})	0.695**<br>(p = 3.3e^{-05})	0.605**<br>(p = 3.1e^{-04})	0.719**<br>(p 1.7e^{-05})	0.797**<br>(p = 1.3e^{-08})	1.000<br>—		
Prediction intervals†	0.300<br>(p = 0.141)	0.479**<br>(p = 0.004)	0.212<br>(p = 0.303)	0.123<br>(p = 0.559)	0.300<br>(p = 0.141)	0.255<br>(p = 0.132)	0.410*<br>(p = 0.015)	1.000<br>—	
$p(d_R - d_O)$‡	0.394*<br>(p = 0.019)	0.557**<br>(p = 7.3e^{-05})	0.358*<br>(p = 0.033)	0.289<br>(p = 0.087)	0.394*<br>(p = 0.019)	0.342*<br>(p = 0.015)	0.532**<br>(p = 1.5e^{-04})	0.713**<br>(p = 2.1e^{-05})	1.000<br>—

Supplementary Table 7. Correlations between replicability forecasts and replication indicators (not preregistered). The table reports Pearson product-moment correlation coefficients ρ between *(i)* final decision market prices and *(ii)* the average prediction survey beliefs and the various replication indicators reported in the main text. p-values are reported in parentheses. Dichotomous replication indicators are marked with a dagger (†), continuous indicators are marked with a double dagger (‡). n = 26 for all correlations. * $p < 0.05$, ** $p < 0.005$; two-sided tests..

	Stat. sig. indicator†	*Relative effect size*‡	*Small telescopes*†	*Meta effect* α = *5%*†	*Meta effect* α = *0.5%*†	BF_{+0}‡	BF_{R0}‡	*Prediction intervals*†	$p(d_R - d_O)$‡
Final market prices	0.505*	0.391*	0.549**	0.625**	0.505*	0.248	0.204	−0.236	0.069
	(p = 0.008)	(p = 0.048)	(p = 0.004)	(p = 0.001)	(p = 0.008)	(p = 0.223)	(p = 0.317)	(p = 0.246)	(p = 0.738)
Avg. prediction survey beliefs	0.476*	0.383	0.505**	0.597**	0.476*	0.284	0.304	−0.236	0.064
	(p = 0.014)	(p = 0.054)	(p = 0.004)	(p = 0.001)	(p = 0.014)	(p = 0.159)	(p = 0.131)	(p = 0.246)	(p = 0.758)

Supplementary Table 8. Survey results. The table reports the average forecasted probability of replication (and the standard deviation of beliefs) for each of the 41 hypotheses. The table also shows the mean survey response to the question of whether the Covid-19 pandemic is expected to impact replication outcomes together with the *t*-statistic and two-sided *p*-value obtained from a one-sample *t*-test ($df = 161$) for each of the 41 hypotheses. As per our pre-analysis plan, all survey results are based on the sample of survey respondents who eventually participated actively in the decision market ($n = 162$). As a reference, the table indicates whether the study was selected for replication (and, if so, whether the hypothesis belongs to the "top-12" or "bottom-12" group (as based on the final decision market prices), or whether it has been selected at random). Furthermore, the absolute prediction error and the Brier score (i.e., the squared prediction error) of the survey forecasts are reported beside an indicator for whether the replication was successful (as per the statistical significance indicator).

			Impact of Covid-19					*Prediction accuracy*	
Study	*Avg. survey belief*	*St. dev. of beliefs*	*Avg. belief*	*t-statistic*	*p-value*	*Selected*	*Replication outcome*	*Abs. pred. error*	*Brier score*
Ames & Fiske[1]	0.689	0.213	0.080	1.798	0.074	top-12	☒	0.689	0.474
Atir & Ferguson[2]	0.425	0.253	−0.012	−0.470	0.639	bottom-12	☒	0.425	0.180
Baldwin & Lammers[3]	0.415	0.234	−0.154	−3.419	0.001	bottom-12	☒	0.415	0.172
Bear et al.[4]	0.575	0.230	0.006	0.229	0.819	—	☐	—	—
Boswell et al.[5]	0.748	0.183	−0.043	−0.927	0.355	top-12	☑	0.252	0.063
Caruso et al.[6]	0.710	0.186	0.025	1.000	0.319	—	☐	—	—
Casella et al.[7]	0.748	0.178	0.019	0.774	0.440	—	☐	—	—
Chao[8]	0.420	0.249	−0.105	−3.135	0.002	bottom-12	☑	0.580	0.336
Cheon & Hong[9]	0.323	0.243	0.117	2.514	0.013	bottom-12	☒	0.323	0.104
Clarkson et al.[10]	0.486	0.256	0.123	2.156	0.033	—	☐	—	—
Cooney et al.[11]	0.751	0.215	0.049	1.576	0.117	top-12	☑	0.249	0.062
Côté et al.[12]	0.420	0.242	−0.006	−0.100	0.920	bottom-12	☒	0.420	0.176
Flesch et al.[13]	0.511	0.236	−0.012	−1.000	0.319	—	☐	—	—

cont'd on next page

			Impact of Covid-19					Prediction accuracy	
Study	*Avg. survey belief*	*St. dev. of beliefs*	*Avg. belief*	*t-statistic*	*p-value*	*Selected*	*Replication outcome*	*Abs. pred. error*	*Brier score*
Genschow et al.[14]	0.393	0.253	−0.043	−1.185	0.238	bottom-12	☑	0.607	0.368
Gheorghiu et al.[15]	0.449	0.262	−0.037	−0.787	0.432	bottom-12	☑	0.551	0.304
Guilbeault et al.[16]	0.524	0.270	0.130	2.534	0.012	random	☒	0.524	0.274
Halevy & Halali[17]	0.757	0.194	0.043	1.404	0.162	top-12	☑	0.243	0.059
Handley et al.[18]	0.475	0.278	−0.093	−2.748	0.007	—	☐	—	—
Hoffman et al.[19]	0.422	0.258	0.043	0.830	0.408	bottom-12	☒	0.422	0.178
Hofstetter et al.[20]	0.701	0.205	0.062	1.550	0.123	top-12	☒	0.299	0.089
Horne et al.[21]	0.538	0.228	−0.568	−4.196	$4.5e^{-05}$	—	☐	—	—
Isley et al.[22]	0.639	0.218	−0.068	−1.181	0.239	—	☐	—	—
Jachimowicz et al.[23]	0.470	0.252	0.062	1.340	0.182	—	☐	—	—
John et al.[24]	0.447	0.264	−0.031	−0.629	0.530	bottom-12	☒	0.447	0.200
Jordan et al.[25]	0.724	0.175	0.000	0.000	1.000	random	☒	0.724	0.524
Jun et al.[26]	0.547	0.229	−0.037	−0.517	0.606	—	☐	—	—
KC et al.[27]	0.499	0.216	0.000	0.000	1.000	—	☐	—	—
Klein & O'Brien[28]	0.818	0.173	0.019	0.556	0.579	top-12	☑	0.182	0.033
Kouchaki & Gino[29]	0.590	0.235	0.025	0.816	0.416	bottom-12	☒	0.590	0.348
Kraus et al.[30]	0.680	0.220	0.068	1.074	0.284	top-12	☑	0.320	0.102
McCall et al.[31]	0.754	0.197	0.389	5.299	$3.8e^{-07}$	top-12	☑	0.246	0.060
Morris et al.[32]	0.738	0.223	0.056	1.892	0.060	top-12	☑	0.262	0.068

cont'd on next page

Study	Avg. survey belief	St. dev. of beliefs	Impact of Covid-19			Selected	Replication outcome	Prediction accuracy	
			Avg. belief	t-statistic	p-value			Abs. pred. error	Brier score
Mummolo[33]	0.522	0.234	0.111	1.681	0.095	—	☐	—	—
Payne et al.[34]	0.527	0.233	0.130	2.572	0.011	—	☐	—	—
Phillips & Cushman[35]	0.728	0.200	−0.037	−1.096	0.275	—	☐	—	—
Rai et al.[36]	0.502	0.252	0.105	1.904	0.059	bottom-12	☑	0.498	0.248
Reeck et al.[37]	0.467	0.223	−0.049	−1.337	0.183	bottom-12	☒	0.467	0.218
Schilke et al.[38]	0.684	0.229	−0.049	−1.156	0.249	—	☐	—	—
Stern et al.[39]	0.66	0.217	0.302	6.005	$1.2e^{-08}$	top-12	☒	0.660	0.436
Vacharkulksemsuk et al.[40]	0.803	0.209	0.037	1.611	0.109	top-12	☑	0.197	0.039
Williams et al.[41]	0.641	0.246	−0.037	−0.801	0.424	top-12	☑	0.359	0.129
